# HLTF Resolves G4s and Promotes G4-Induced Replication Fork Slowing to Maintain Genome Stability

**DOI:** 10.1016/j.molcel.2024.07.018

**Published:** 2024-08-13

**Authors:** Gongshi Bai, Theresa Endres, Ulrike Kühbacher, Valentina Mengoli, Briana H. Greer, Emma M. Peacock, Matthew D. Newton, Tyler Stanage, Maria Rosaria Dello Stritto, Roxana Lungu, Magdalena P. Crossley, Ataya Sathirachinda, David Cortez, Simon J. Boulton, Petr Cejka, Brandt F. Eichman, Karlene A. Cimprich

**Affiliations:** 1 Stanford University, Department of Chemical & Systems Biology, Stanford, CA 94305; 2 Institute for Research in Biomedicine, Università della Svizzera italiana, Bellinzona, Switzerland; 3 Vanderbilt University, Department of Biological Sciences, Nashville, TN 37232; 4 DSB Repair Metabolism Laboratory, The Francis Crick Institute, 1 Midland Road, London NW1 1AT, UK; 5 Vanderbilt University, Department of Biochemistry, Nashville, TN 37232

## Abstract

G-quadruplexes (G4s) form throughout the genome and influence important cellular processes. Their deregulation can challenge DNA replication fork progression and threaten genome stability. Here, we demonstrate an unexpected role for the dsDNA translocase HLTF in responding to G4s. We show that HLTF, which is enriched at G4s in the human genome, can directly unfold G4s *in vitro* and uses this ATP-dependent translocase function to suppress G4 accumulation throughout the cell cycle. Additionally, HLTF and MSH2, a component of MutS heterodimers which bind G4s, act independently to suppress G4 accumulation, to restrict alternative lengthening of telomeres and to promote resistance to G4 stabilizing drugs. In a discrete but complementary role, HLTF restrains DNA synthesis when G4s are stabilized by suppressing PrimPol-dependent repriming. Together, the distinct roles of HLTF in the G4 response prevent DNA damage and potentially mutagenic replication to safeguard genome stability.

## Introduction

DNA-damaging agents, protein-DNA complexes, and noncanonical nucleic acid secondary structures can threaten genome stability.^1–4^ Among the latter are G-quadruplexes (G4s), nucleic acid structures that form upon the stacking of planar guanidine tetrads.^5,6^ G4s form in G-rich genomic regions,^5,7,8^ and play physiological roles in transcription,^9–11^ telomere homeostasis,^12–15^ DNA replication initiation,^16^ and epigenetic inheritance.^17,18^ Deregulated G4 formation and stabilization, however, can inhibit DNA replication^19–21^ and transcription,^22–24^ challenging genome stability.^25–27^ Indeed, G4-forming motifs are determinants of mutagenesis in the cancer genome.^28^ To mitigate the adverse effects of G4s, cells utilize several proteins to resolve these structures, including helicases and ssDNA binding proteins.^6,29^ Additionally, the MSH2-MSH6 (MutSα) and MSH2-MSH3 (MutSβ) complexes, whose canonical role is in DNA mismatch repair,^30^ can bind G4s and regulate their stability.^31–34^

When deregulated, G4s pose a barrier to replication fork progression^19–21,35^ and can cause DNA replication stress,^36^ ultimately causing DNA double-strand breaks (DSBs).^37–41^ DNA damage tolerance (DDT) pathways promote the bypass of replication fork barriers, allowing their subsequent repair or resolution and promoting replication stress tolerance.^41^ DDT pathways include replication fork reversal, repriming of DNA synthesis using the alternative primase-polymerase (PrimPol) and translesion synthesis (TLS).^41^ While fork reversal is considered a high-fidelity form of DDT, the other pathways can be error-prone.^42,43^

First identified as a transcription factor,^44,45^ helicase-like transcription factor (HLTF) regulates several forms of DDT. HLTF associates with replication forks^46–48^ and helps to control the choice between DDT pathways by ubiquitinating PCNA using its RING-domain.^49–52^ It also reverses replication forks using its ATP-dependent dsDNA translocase activity and its HIRAN domain.^47,53–56^ Importantly, this action slows fork progression and restrains PrimPol-mediated DNA synthesis upon replication stress.^57^

HLTF also mediates other types of DNA transactions. It promotes efficient nucleotide excision repair by evicting the lesion-containing oligonucleotide using both its translocase activity and HIRAN domain.^58^ HLTF also has a strand invasion activity that depends on its ATPase domain.^59^ Furthermore, it can displace ssDNA in a triplex structure by translocating on dsDNA in an ATP-dependent manner.^53^ Whether HLTF uses its ATP-dependent translocase activity to act on other types of DNA structures is unknown.

Consistent with its genome maintenance functions, HLTF is a tumor suppressor.^60^ Knockout of HLTF in mice causes genome instability and susceptibility to intestinal carcinogenesis.^61^ Moreover, HLTF silencing is frequently observed in colorectal and gastric cancers^62,63^ and is associated with poor prognosis.^64^ Understanding HLTF’s functions may therefore reveal vulnerabilities associated with HLTF-deficient cancers.

Here, we uncover an unexpected function for HLTF in maintaining genome stability through G4 regulation. We find that HLTF binds chromatin in a transcription-dependent manner and at G4s, and that it suppresses the formation of G4 structures throughout the cell cycle. G4 suppression by HLTF requires its ATPase activity in cells and may be attributed to its ability to directly resolve G4s using its dsDNA translocase activity. Interestingly, HLTF-deficient cells also tolerate replication stress caused by G4 stabilization using PrimPol, yet are sensitive to drugs that stabilize G4 structures. Taken together, our studies reveal a previously unknown mechanism for G4 resolution and show that HLTF loss helps cells to tolerate the formation of G4s, increasing the probability of DNA damage and mutagenesis.

## Results

### HLTF and MSH2 bind chromatin in a reciprocal and cell cycle-independent manner

**Figure 1:**
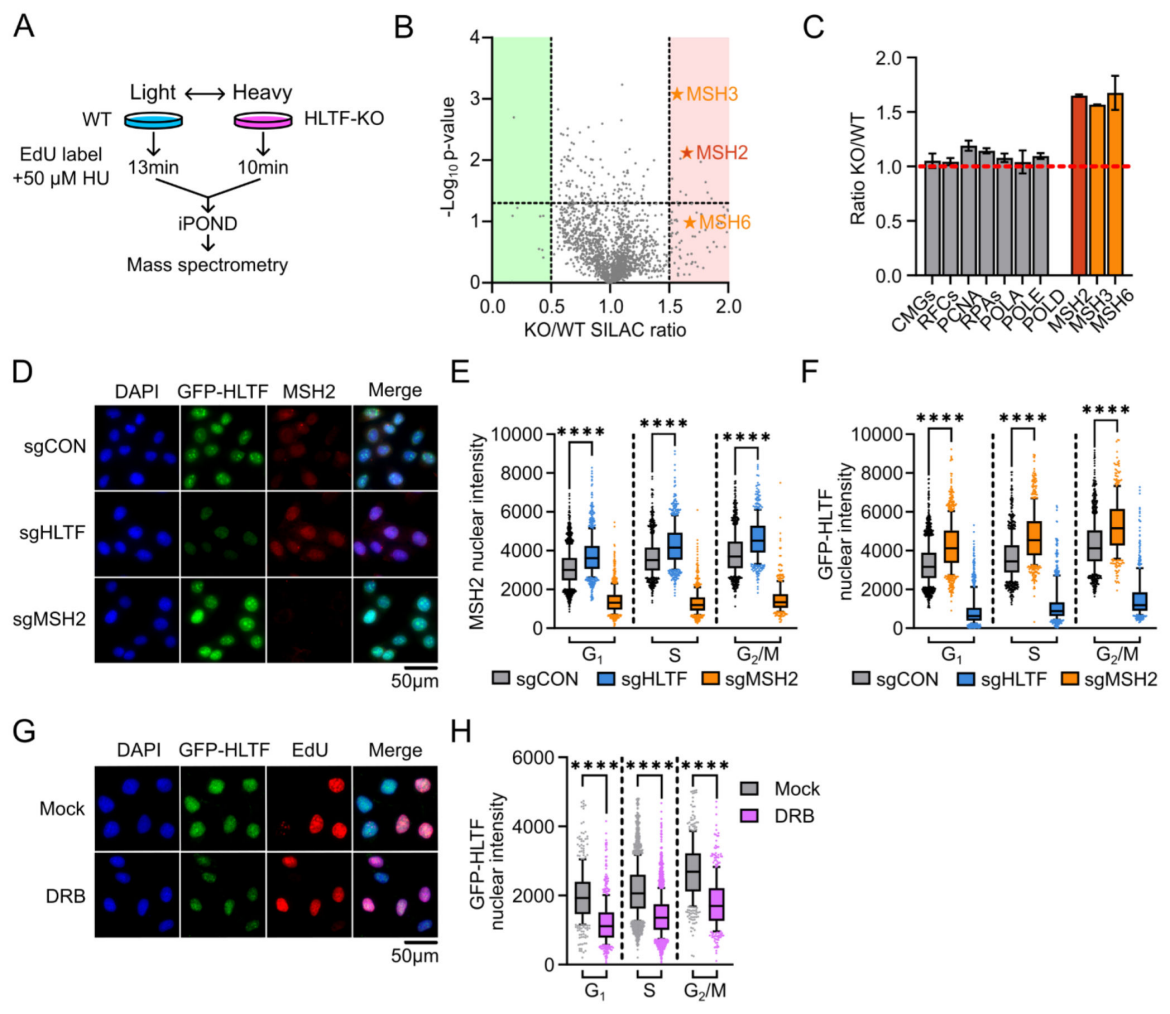
The cell cycle independent regulation of HLTF chromatin binding by MSH2 and transcription. A. Schematic of iPOND-SILAC-MS. “Heavy” and “light” amino acids labeling of WT or HLTF-KO cells was reversed for the two biological repeats. B. Volcano plots for proteins identified by iPOND-SILAC-MS. p-values are calculated based on two biological repeats. Protein abundance changes with at least 50% decrease (green) or increase (red) are highlighted. Significance cutoff for protein enrichment was set at p=0.05 (horizontal dotted line). C. HLTF-KO/WT SILAC ratio for proteins from B. For CMGs, RFCs, RPAs, and replicative polymerases, mean ± SEM is calculated from the normalized SILAC ratio of each subunit comprising the complex. For MSH2, 3 and 6, the normalized SILAC ratio is used to calculate the mean ± SD (n=2). D. Representative immunofluorescence (IF) images of U2OS cells expressing GFP-HLTF after sgRNA-mediated knockdown. GFP-HLTF is detected using a GFP antibody. E. Mean intensity of chromatin-bound MSH2 as shown in D. See also Figure S1E. F. Mean intensity of chromatin-bound GFP-HLTF as shown in D. See also Figure S1G. G. Representative IF images of U2OS cells expressing GFP-HLTF after mock or DRB treatment (100 µM, 4h). GFP-HLTF is detected using a GFP antibody. H. Mean intensity of chromatin-bound GFP-HLTF from G. See also Figure S1K. Mann-Whitney tests were performed for all data shown in this figure.

We previously showed that HLTF restrains replication fork progression in response to DNA replication stress.^47,57^ To understand this phenotype, we examined the proteomic composition of nascent DNA using isolation of proteins on nascent DNA combined with SILAC quantitative mass spectrometry (iPOND-SILAC-MS)^46,65^ in wild-type (WT) and HLTF-knock-out (HLTF-KO) HEK293T cells treated with hydroxyurea (HU) (Figure 1A; Figure S1A). Analysis of the proteins enriched on chromatin (Figure 1B, Supplemental Tables 1&2) revealed that core replisome components were equally enriched on nascent DNA in the two conditions (Figure 1C). By contrast, we observed over 50% increase in the enrichment of several proteins involved in DNA mismatch repair (MMR) on chromatin isolated from HLTF-KOs (Figure 1B, C).

MSH2 interacts with MSH3 or MSH6 to form the MutSα or β complexes, respectively. These proteins bind DNA during S/G_2_ to promote the repair of mismatches formed during DNA synthesis.^30^ Hence, we hypothesized that they might act on mismatches that result from the action of PrimPol and TLS polymerases in HLTF’s absence.^57^ To test this idea and validate our iPOND data, we analyzed MSH2 chromatin binding using quantitative image-based cytometry (QIBC) in pre-extracted U2OS cells expressing endogenously tagged GFP-HLTF (Figure S1B) and pulse-labeled with EdU.^58^ We observed increased association of MSH2 with chromatin upon knocking down HLTF (Figure 1D; Figure S1C, D). Unexpectedly, this increase was observed not only in S and G_2_/M phase cells but also in G_1_ cells (Figure 1E; Figure S1E). This suggests that HLTF suppresses the binding of MSH2 to chromatin throughout the cell cycle, inconsistent with the idea that these proteins only function in post-replicative repair.

Next, we asked if MSH2 loss alters HLTF’s association with chromatin. Surprisingly, knockdown of MSH2 in either the GFP-HLTF cell line, or in wild-type U2OS cells, increased HLTF chromatin binding in all cell cycle phases (Figure 1D, F; Figure S1F-J). Hence, HLTF and MSH2 can suppress the interaction of the other with chromatin throughout the cell cycle.

Given HLTF’s potential link to transcription,^44,45^ we asked if its chromatin binding is transcription-dependent. Treatment with the transcription inhibitor 5,6-dichlorobenzimidazole (DRB) reduced HLTF chromatin binding in all cell cycle phases (Figure 1G, H; Figure S1K, L). These results indicate that HLTF interacts with chromatin in a transcription-dependent and replication-independent manner that is increased when MSH2 is lost. They also suggest that HLTF and MSH2 interact with chromatin in a reciprocal manner.

### HLTF prevents the accumulation of G4 structures

**Figure 2:**
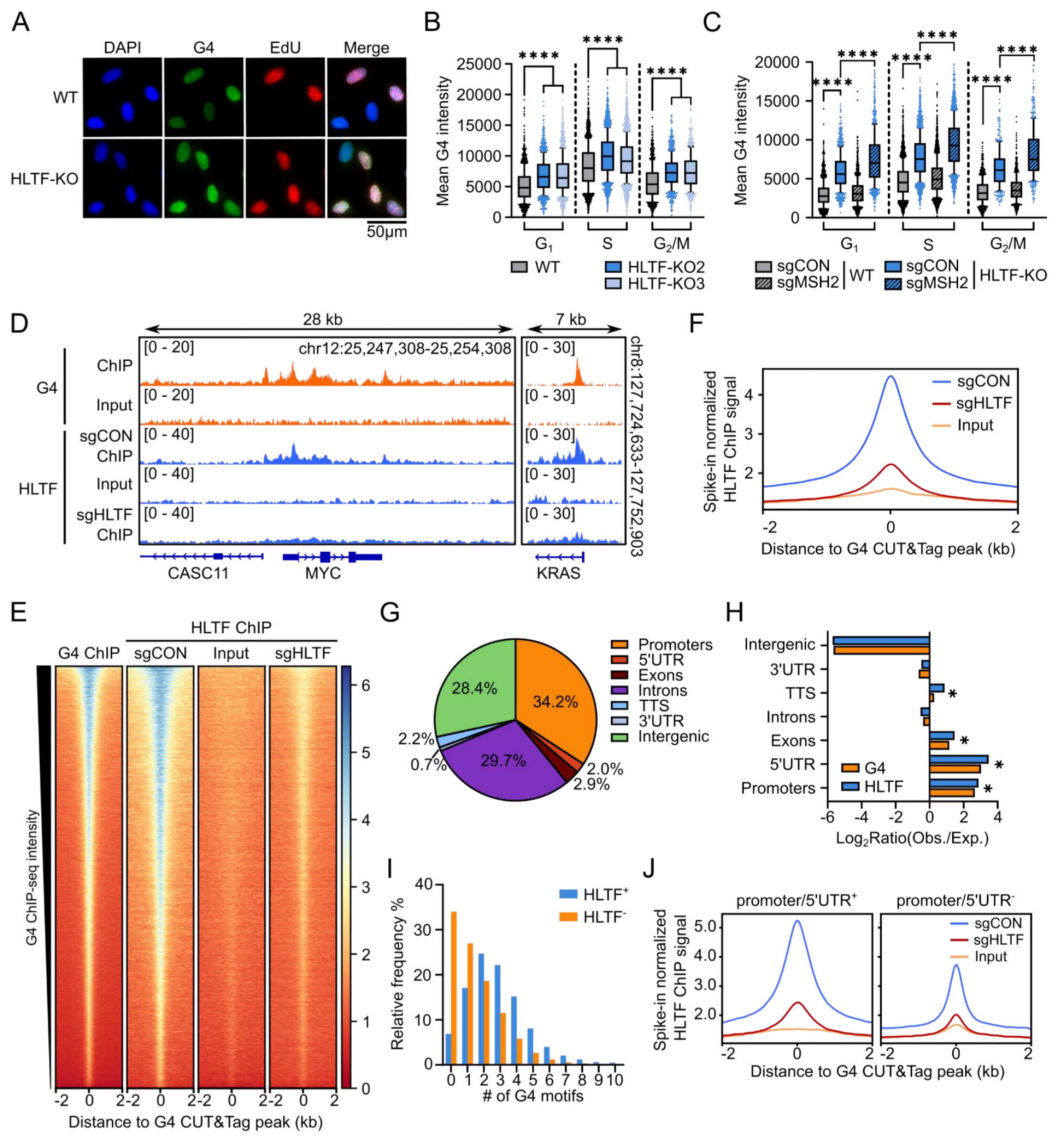
HLTF suppresses G4 accumulation in cells and is enriched at G4 structures in the human genome. A. Representative IF images in WT and HLTF-KO U2OS cells. G4s are detected using the 1H6 antibody. B. Mean G4 intensity from A. See also Figure S2C. C. Mean G4 intensity in WT and HLTF-KO U2OS cells after sgRNA-mediated knockdown. See also Figure S2G. Mann-Whitney tests were performed in B and C. D. Representative browser tracks of G4 ChIP-seq (GSE162299) and HLTF ChIP-seq at the *MYC* and *KRAS* loci. E. Heatmaps showing ChIP-seq coverage for G4 and HLTF at G4 CUT&Tag peaks in U2OS cells (n=35,104) (GSE181373). The x-axis represents the distance from the peak in kb. Heatmaps were sorted by G4 ChIP-seq signal intensity for all ChIP-seq samples. Spearman correlation coefficient between G4 and HLTF sgCON ChIP-seq signal was 0.74. F. Aggregate plot showing HLTF ChIP-seq coverage (y-axis) relative to the distance (x-axis, in kb) from G4 CUT&Tag peaks, related to E. G. Distribution of HLTF ChIP-seq peaks within each genomic compartment. TTS, transcription termination site. H. Relative enrichment of G4 CUT&Tag and HLTF ChIP-seq peaks within each genomic compartment. * indicates compartments where both G4 and HLTF showed significant enrichment. I. Frequency distribution of the number of G4 motifs within G4 CUT&Tag peaks. G4 peaks are segregated based on overlap with HLTF ChIP-seq peaks: G4 peaks that overlap with HLTF peaks are HLTF^+^ or otherwise HLTF^-^. See also Figure S2J. J. Aggregate plot showing HLTF ChIP-seq coverage (y-axis) relative to the distance (x-axis, in kb) from G4 CUT&Tag peaks. G4 peaks are segregated based on whether they are within the promoter or 5’UTR (G4 in promoter/5’UTR or promoter/5’UTR^+^, n=17,620; G4 out of promoter/5’UTR or promoter/5’UTR^-^, n=17,484). Promoters are identified as ±1kb of the annotated transcription start site. See also Figure S2K.

Next, we investigated the function of HLTF and MSH2 on chromatin. Nucleic acid secondary structures such as G4s and RNA-DNA hybrids can form co-transcriptionally.^66–68^ In addition to binding DNA mismatches,^30^ MutSα and β complexes can interact with some nucleic acid secondary structures,^69–73^ including G4s.^31–34^ Given that HLTF’s chromatin binding is transcription-dependent and affected by the presence of MSH2, we asked if HLTF controls G4 levels in cellular DNA. We observed an increase in G4s in HLTF-KO cell lines using two cell types and two antibodies raised against G4-structured DNA (Figure 2A, B; Figure S2A-D). Similarly, transient HLTF knockdown increased G4s (Figure S2E). G4 stabilization with pyridostatin (PDS) also increased HLTF chromatin binding (Figure S2F). In each case, the increase in G4s or HLTF was observed in all cell cycle phases.

We then asked whether knocking down MSH2 increased G4s. We observed a modest increase in the G4 signal when MSH2 was knocked down in wild-type cells and a synergistic increase in an HLTF-KO U2OS cell line (Figure 2C; Figure S2G). Taken together, our data suggest that HLTF and MSH2 act in independent pathways to suppress G4 accumulation throughout the cell cycle.

### HLTF binds to G4-containing loci throughout the genome

The ability of HLTF to suppress G4 accumulation and the increase in its chromatin binding following PDS treatment indicate that HLTF might act at G4s in the genome. To test this hypothesis, we performed spike-in normalized chromatin immunoprecipitation-sequencing (ChIP-seq)^74^ in the GFP-HLTF U2OS cell line. First, we examined the HLTF ChIP-seq signal at previously identified G4-forming sites (Figure 2D, E).^11,75^ We found that HLTF binds to G4s throughout the genome and that its enrichment on DNA correlates with G4 formation (Figure 2E, F). Additionally, we found that HLTF is specifically enriched at those motifs that actually form G4s in cells and not at those that only form *in vitro* (Figure S2H).^76^ Hence, HLTF is enriched at *bona fide* G4 structures in cells. We also investigated MSH2 enrichment at G4s, using previously published datasets from mouse embryonic stem cells (E14).^77,78^ Like HLTF, MSH2 is enriched at G4s (Figure S2I).

Next, we identified HLTF binding sites in the genome, calling 7,614 peaks. HLTF peaks are predominantly found at the beginning of genes, including in promoters and 5’UTRs (Figure 2G, H), a distribution similar to that observed for G4s (Figure 2H).^9^ Moreover, 56% of HLTF peaks overlap with G4-forming sites (Figure S2J) and were more likely to contain multiple G4-forming motifs (Figure 2I).^76^ We also asked if HLTF’s enrichment at G4s is due to its binding to transcriptional regulatory regions. We found that HLTF also binds to G4s outside of these regions and that binding still correlates with G4 levels (Figure 2J; Figure S2K). Taken together, these data demonstrate that HLTF is enriched at G4s throughout the genome and its binding is a function of G4 density, consistent with its role in regulating G4 levels.

### HLTF is enriched at RNA-DNA hybrids stabilized by G4s

**Figure 3:**
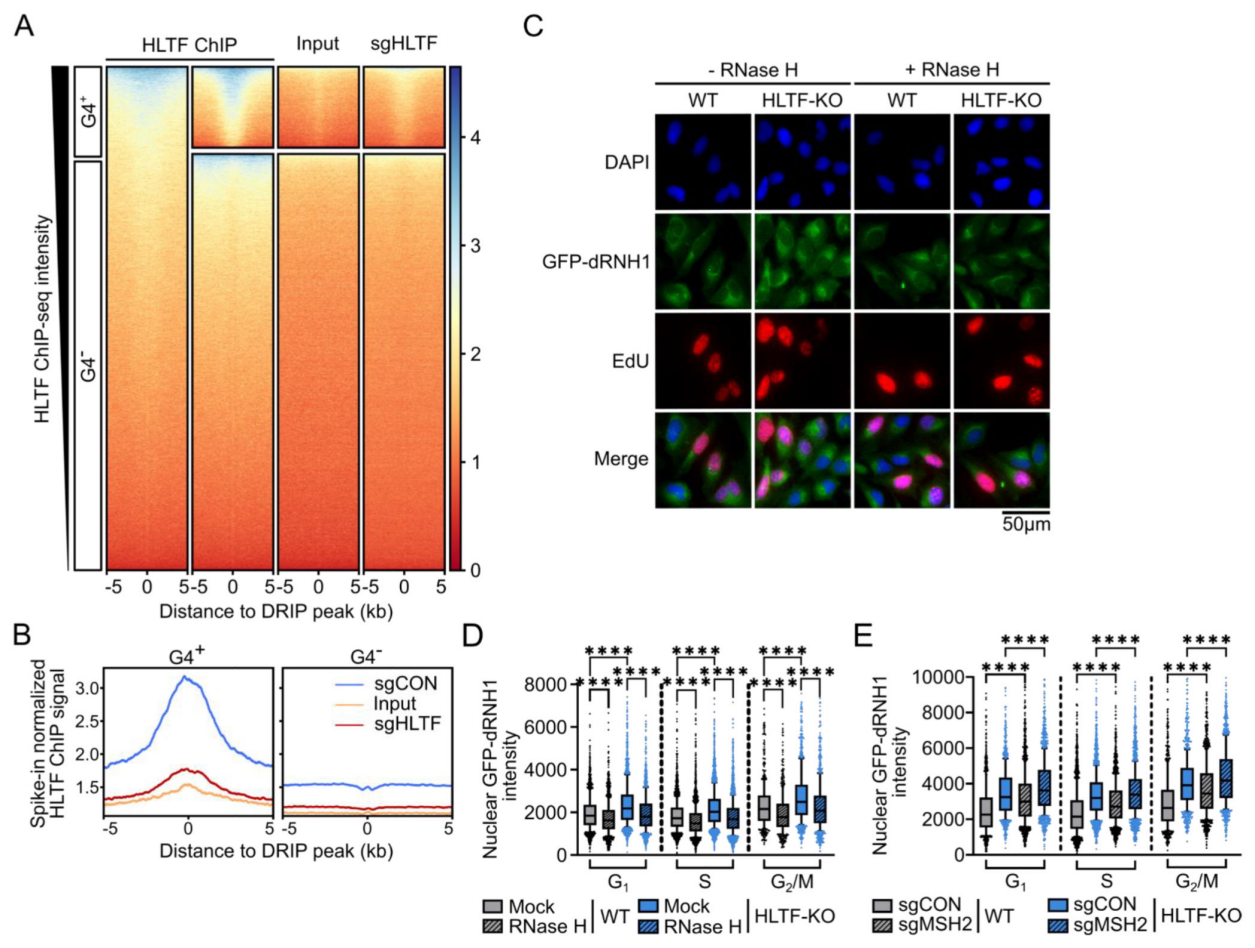
HLTF is enriched at RNA-DNA hybrids stabilized by G4s. A. Heatmaps showing ChIP-seq coverage for HLTF at RNA-DNA hybrid peaks identified in U2OS cells by DRIP-seq (GSE115957). The x-axis represents the distance from the DRIP peak in kb. DRIP peaks are segregated based on whether they overlap with G4 peaks identified by G4 CUT&Tag: DRIP peaks that overlap with G4 peaks are G4^+^(n=11,499) or otherwise G4^-^ (n=59,090). B. Aggregate plot showing HLTF ChIP-seq coverage (y-axis) relative to the distance in kb (x-axis) from DRIP peaks. Related to A. See also Figure S3A. C. Representative IF images of RNA-DNA hybrids in WT and HLTF-KO U2OS cells after mock or RNase H digestion. Hybrids are detected using purified, recombinant GFP-dRNH1. D. Mean nuclear hybrid intensity, as shown in C. See also Figure S3D. E. Mean nuclear hybrid intensity in WT and HLTF-KO U2OS after sgRNA transfection. See also Figure S3E. Mann-Whitney tests were performed in this figure.

G4s can co-occur with RNA-DNA hybrids at transcriptionally active regions and at telomeres,^15,79^ with each structure stabilizing the other.^66,79,80^ Hence, we assessed HLTF enrichment at RNA-DNA hybrids previously identified by DRIP-seq in U2OS cells.^26^ HLTF’s enrichment is modest at these sites (Figure 3A). Interestingly, however, its enrichment is substantial at the 15% of hybrid sites that overlap with G4s (Figure 3A, B; Figure S3A). HLTF enrichment is also stronger at the subset of G4s that overlap with RNA-DNA hybrids (Figure S3B). A similar analysis of the MSH2 ChIP-seq signal at G4s and RNA-DNA hybrids in mESCs revealed that MSH2 is only enriched at hybrids that co-occur with G4s (Figure S3C). These observations suggest that HLTF and MSH2 interact with RNA-DNA hybrids indirectly and through their action on G4s.

To further test the relationship between HLTF and RNA-DNA hybrids, we measured hybrid accumulation.^81^ We observed a small but significant RNaseH-reversible increase in hybrids in HLTF-KOs (Figure 3C, D; Figure S3D). MSH2 knockdown alone modestly increased hybrids, and its knockdown in HLTF-KOs led to a further increase in all cell cycle phases (Figure 3E; Figure S3E). We also found that RNaseH expression reduced global HLTF chromatin binding (Figure S3F, G). Furthermore, using ChIP-qPCR we found that HLTF binding is specifically reduced at G4s stabilized by an RNA-DNA hybrid (Figure S3H). Taken together, our data indicate that HLTF’s effect on hybrids is restricted to those stabilized by G4s.

### HLTF suppresses G4 accumulation in an ATPase-dependent manner

**Figure 4:**
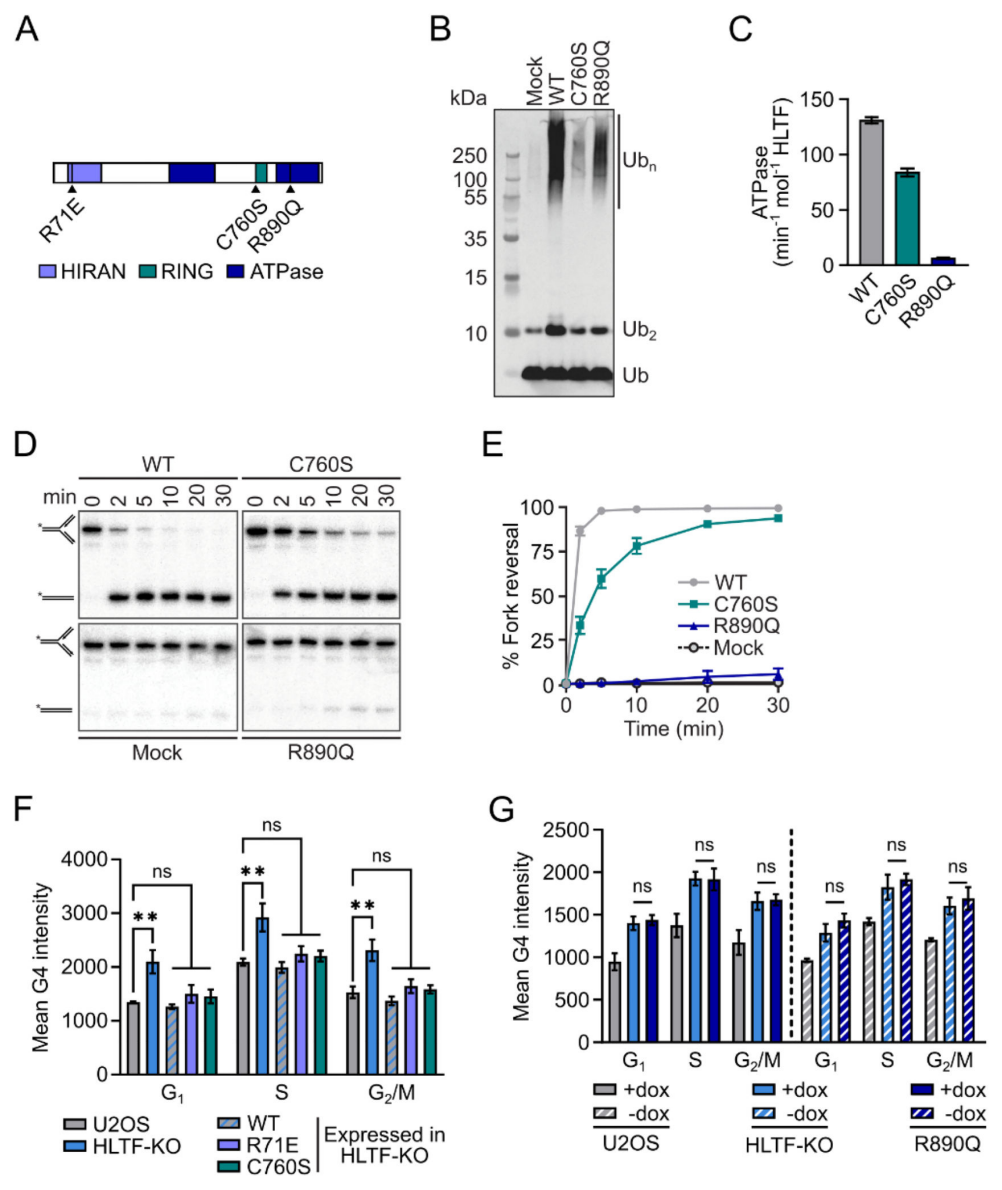
HLTF suppresses G4s in an ATPase-dependent manner in cells. A. Schematic of HLTF domain structures. Arrowheads represent position of mutations. B. Western blot of HLTF-dependent Ub chain formation by UBC13/MMS2 using a ubiquitin antibody. C. ATPase rates of HLTF WT and mutant proteins measured using an NADH-coupled assay. Rates were corrected for background NADH decomposition in a no-enzyme control. Data are plotted as the mean ± SD (n=3). See also Figure S4B. D. Denaturing PAGE showing the separation of DNA substrate (fork) and product (duplex), as a measurement of the *in vitro* fork reversal activity of HLTF WT and mutant proteins. E. Quantification of the *in vitro* fork reversal activity of HLTF WT and mutant proteins. Related to D. Data are plotted as the mean ± SD (n=3). F. Mean G4 intensity in U2OS WT and HLTF-KO cells constitutively expressing HLTF WT or mutant proteins. Two clones of each cell line were analyzed separately. The median of the mean G4 intensity of the two clones is averaged to calculate the mean ± SEM (n=3). One-way ANOVA was performed followed by Dunnett’s test. G. Mean G4 intensity in U2OS WT and HLTF-KO cells inducibly expressing HLTF R890Q mutant, after dox induction (24 h, 500 ng/mL). Two clones of R890Q expressing HLTF-KO cell lines were analyzed separately. The median of the mean G4 intensity of the two clones is averaged to calculate the mean ± SEM (n=3). T-test was performed.

Next, we sought to investigate the role of HLTF’s different domains (Figure 4A) in G4 suppression using parental U2OS and HLTF-KO cells expressing either WT or HLTF separation-of-function mutations. We previously generated a HIRAN domain mutant (R71E) that prevents 3’-OH DNA end binding and fork remodeling, but does not alter HLTF’s ATPase and ubiquitin ligase activities.^47,57^ For this study, we generated two new mutants. A RING domain mutation (C760S)^82^ abolished HLTF’s ubiquitin ligase activity but retained fork reversal and ATPase activity *in vitro* (Figure 4B-E; Figure S4A). We also generated a new ATPase-defective mutant (R890Q) since we could not stably express other ATPase mutants in cells. To do so, we modified residue in HLTF analogous to one needed for the ATPase activity of SMARCAL1 (Figure S4B), a related fork remodeler^83^ and confirmed that this mutant retained its ubiquitin ligase activity (Figure 4B-E; Figure S4A).

As expected, the expression of WT HLTF in HLTF-KO cells reduced G4 levels to those found in the parental U2OS cell line in all cell cycle phases. Similarly, expression of either the R71E or C760S mutant suppressed G4 accumulation (Figure 4F; Figure S4C). By contrast, the ATPase mutant, while still able to bind chromatin (Figure S4D, E), failed to suppress G4s when expressed in HLTF-KOs (Figure 4G). These data indicate that HLTF requires its ATPase domain to suppress G4 accumulation in cells. Thus, HLTF’s ability to prevent G4 accumulation is unlikely related to its effects on replication fork reversal or PCNA ubiquitination, which require the HIRAN^47,55^ and ubiquitin ligase^49,52^ functions, respectively.

### HLTF promotes ATP-dependent G4 unfolding in double-stranded DNA

**Figure 5:**
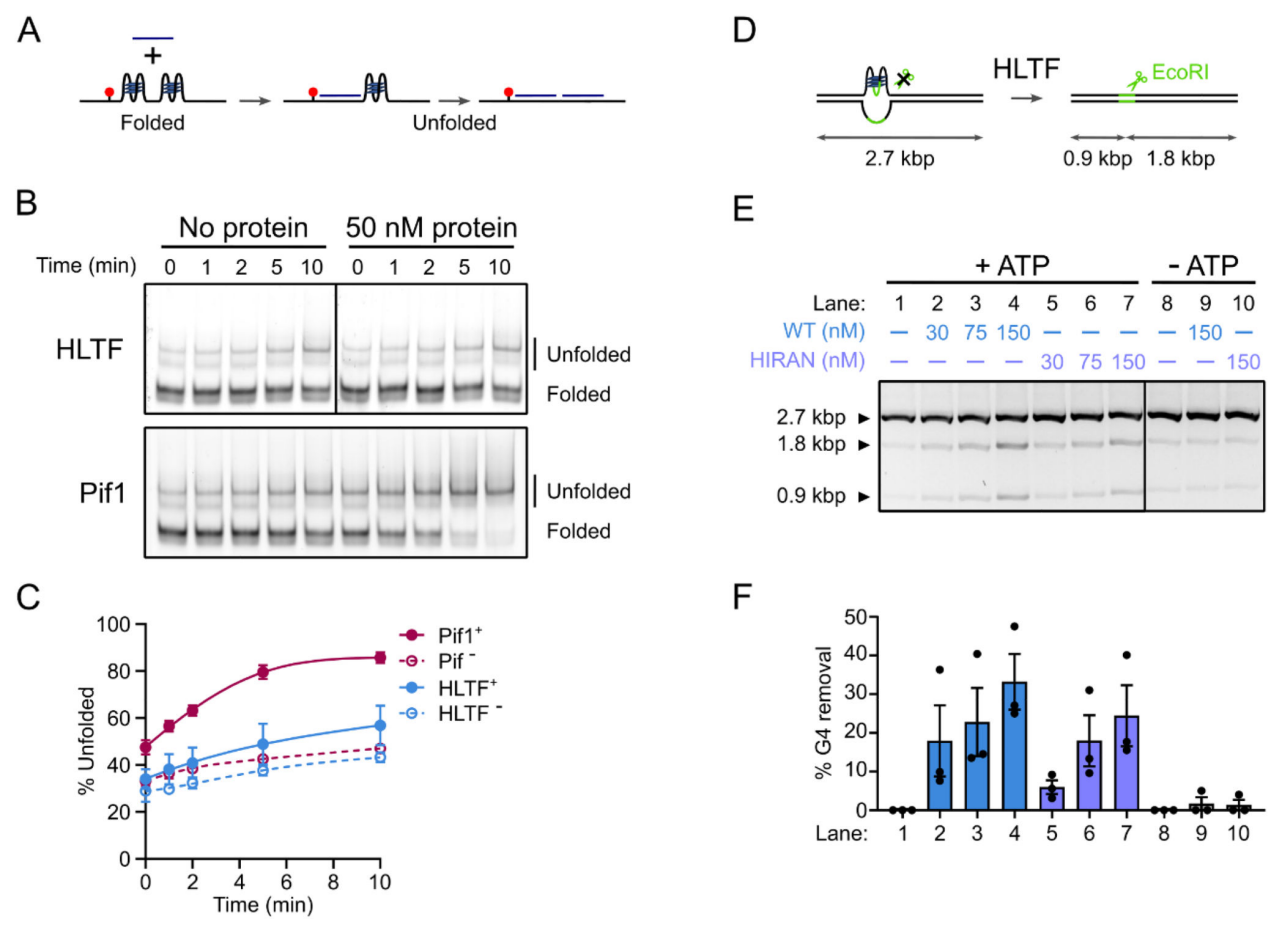
HLTF promotes ATP-dependent G4 unfolding in dsDNA. A. Schematic of ssDNA G4 unfolding assay. B. Native gel images showing ssDNA G4 unfolding by HLTF (top) or *S. cerevisiae* Pif1 (bottom). C. Quantification of ssDNA G4 unfolding by HLTF or *S. cerevisiae* Pif1. Related to B. D. Schematic of dsDNA G4 unfolding assay. E. Representative native gel image showing the dsDNA G4 unfolding by HLTF WT or a HIRAN (N90A,N91A) mutant. DNA substrate in all lanes was digested with EcoRI. F. Quantification of dsDNA G4 unfolding by HLTF WT or a HIRAN (N90A,N91A) mutant. Lane numbers correspond to those shown in E. Data is presented as mean ± SEM (n=3).

Next, we asked whether HLTF can directly unfold or specifically bind G4s *in vitro*. We first tested its activity on a single-stranded oligonucleotide containing a pair of G4s. G4 unfolding was assessed by monitoring the ability of a complementary ssDNA probe to bind the unfolded G4 sequence (Figure 5A). In the absence of HLTF, a modest level of spontaneous unfolding was observed. Addition of the G4 stabilizer Phen-DC3 prevented spontaneous unfolding as expected (Figure S5A, B). Inclusion of HLTF in this reaction had no effect, while the known G4 helicase Pif1, promoted unfolding (Figure 5B, C). Furthermore, we observed no specific binding of HLTF to the G4-containing ssDNA relative to a non-G4-forming control (Figure S5C, D). These findings suggest that HLTF cannot specifically bind to or unwind G4s in the context of ssDNA.

Unlike the Pif1 helicase, HLTF is a dsDNA translocase.^53^ Thus, we reasoned that HLTF might promote G4 resolution in dsDNA by reannealing the DNA strands as it translocates through the structure. To test this, we generated a G4 structure in the context of dsDNA. To monitor G4 unfolding, we introduced an EcoRI cut site into a loop of the G4 forming sequence (Figure 5D). G4 formation in the dsDNA was validated upon addition of PDS, which reduced sensitivity to EcoRI and increased sensitivity to T7 endonuclease I (Figure S5E, F). Importantly, upon addition of wild-type HLTF, we observed increased EcoRI sensitivity (Figure 5E, F; Figure S5G, H). This indicates that HLTF directly unfolds the G4 structure. Similar results were observed with the HIRAN mutant. Moreover, this activity was ATP-dependent (Figure 5E, F). Taken together, these data suggest that HLTF can directly resolve a G4 formed in dsDNA in an ATP-dependent manner.

### HLTF suppresses ALT activity in an ATPase-dependent manner

Next, we asked if HLTF and/or MSH2 suppress alternative lengthening of telomeres (ALT) activity, which can increase upon the stabilization of G4s and hybrids,^15,67,68,84–87^ and which some MutS homologue proteins have been shown to suppress.^33,88^ As a first test of this idea, we assessed the formation of large RPA foci, which can be observed at telomeres undergoing ALT.^89^ Loss of either HLTF or MSH2 led to increased formation of these foci in ALT-positive U2OS cells, with loss of both factors causing a synergistic increase. PDS treatment further increased RPA focus formation in all of these conditions (Figure S6A, B). By contrast, ALT-negative RPE1 cells did not exhibit a significant increase in RPA focus formation after similar treatments (Figure S6C, D). Importantly, ~90% of the RPA foci colocalized with telomeres in WT and HLTF-KO cells, with more telomere-positive RPA foci observed in HLTF-KOs (Figure S6E-G). Telomeres in HLTF-KOs also exhibited increased *γ*H2AX upon PDS treatment, indicating HLTF suppresses DNA damage at ALT telomeres when G4s are stabilized (Figure S6H).

**Figure 6:**
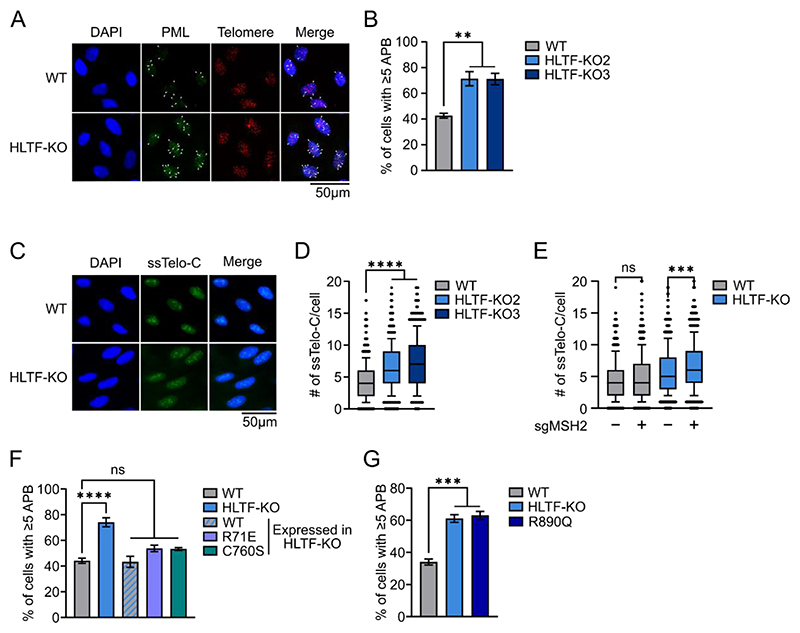
HLTF suppresses ALT activity in an ATPase-dependent manner. A. Representative IF images of ALT-associated PML body (APB) detection in WT and HLTF-KO U2OS cells. Arrowheads mark APBs. B. Percentage of cells with at least five APBs. Related to A. Data are represented as mean ± SEM (n = 3). C. Representative IF images of *in situ* ssTelo-C foci detection in WT and HLTF-KO U2OS cells. D. Quantification of ssTelo-C foci/cell (n=3). Related to C. E. Quantification of ssTelo-C foci/cell (n=4) in WT and HLTF-KO U2OS cells, after transfection with the indicated sgRNA. F. Percentage of cells with at least 5 APBs in WT and HLTF-KO U2OS cells, and HLTF-KO cells expressing HLTF WT, R71E or C760S mutant proteins. Data are represented as mean ± SEM (n ≥ 3). G. Percentage of cells with at least 5 APBs after dox induction (500 ng/mL, 24 h) in WT and HLTF-KO U2OS cells, and HLTF-KO cells expressing the R890Q mutant. Data are represented as mean ± SEM (n = 3). All statistical tests in this figure are one-way ANOVA followed by Dunnett’s test.

Next, we examined several markers of ALT. Telomeres associate with promyelocytic leukemia (PML) protein during ALT to form ALT-associated PML bodies (APBs).^90^ We observed increased formation of APBs in HLTF-KO cells compared to the parental U2OS cells (Figure 6A, B). We also monitored *in situ* ALT-specific single-stranded telomeric C-rich DNA (ssTelo-C), an independent marker for ALT.^91^ ssTelo-C formation also increased in HLTF-KO cells (Figure 6C, D). Although there was no significant effect of MSH2 loss on ssTelo-C formation, consistent with published findings,^33^ simultaneous loss of HLTF and MSH2 further enhanced ALT activity (Figure 6E). Finally, we evaluated APB formation in our HLTF mutant cell lines. We found that the expression of WT, R71E, or C760S mutants suppressed APB formation (Figure 6F), whereas the R890Q mutant did not (Figure 6G). Altogether, these data are consistent with the idea that HLTF reduces ALT through its ability to resolve G4s.

### HLTF restrains replication fork progression in response to G4 stabilization

**Figure 7:**
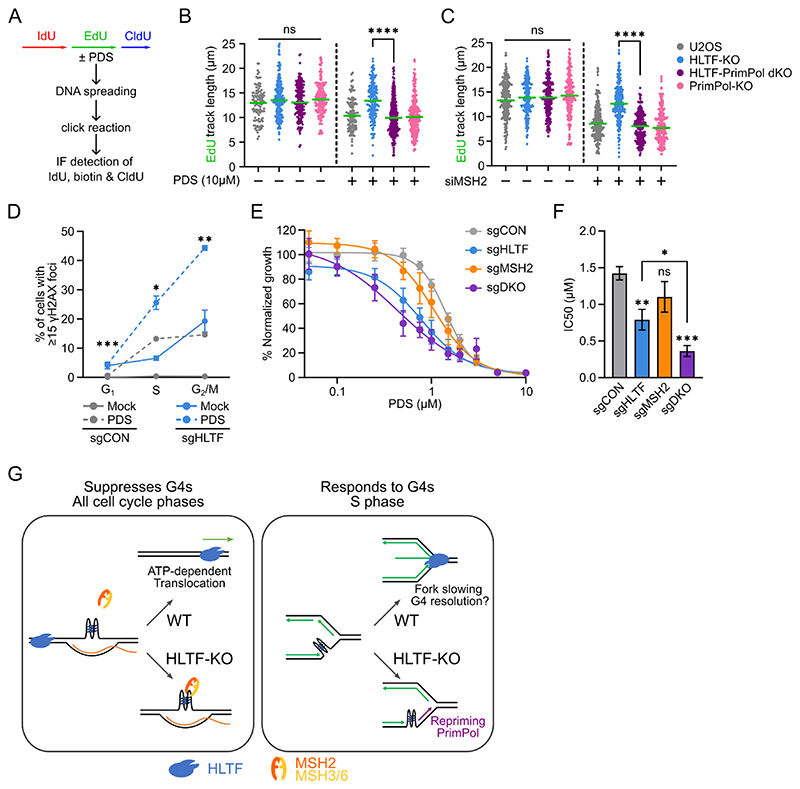
HLTF restrains replication fork progression and protects cells from DNA damage and growth defects in response to G4-stabilization. A. Experimental setup for replication fork progression assay. B. EdU tract lengths (n=3) in mock or PDS-treated U2OS cells. C. EdU tract lengths (n=3) in U2OS cells 72 h after siRNA transfection. In B & C, line represents the median. ns, not significant, by Kruskal-Wallis test; **** p < 0.0001, by Mann-Whitney test. D. Percentage of RPE1 cells with at least 15 γH2AX foci with mock or PDS treatment (5 μM, 24 h) after sgRNA transfection. Data are represented as mean ± SEM (n=3). T-test results comparing the sgCON and sgHLTF PDS-treated samples are shown. E. PDS-response curve in RPE1 cells transfected with the indicated sgRNAs. sgRNAs against both HLTF and MSH2 are transfected in the sgDKO sample. Data are represented as mean ± SEM (n=4). F. IC50 of RPE1 cells after sgRNA transfection and PDS treatment. Related to E. Data are represented as mean ± SEM (n=4). ** p < 0.01; *** p < 0.001; ns, not significant, by one-way ANOVA followed by Dunnett’s test compared to sgCON; * p < 0.05 by t-test. G. Proposed model for the dual roles of HLTF in regulating G4s to maintain genome stability. In its first role (left), HLTF acts in all cell cycle phases and uses its ATPase-dependent dsDNA translocase activity to travel on dsDNA. Upon encounter of the G4, the translocation activity of HLTF may destabilize the structure, allowing the structure forming sequence to reanneal to its complementary strand. MutS complexes can also bind and regulate G4s in a distinct pathway that is independent of HLTF. In a second role (right), HLTF slows DNA synthesis in response to G4 stabilization. G4s may be resolved by other resolution factors as fork reversal occurs. In HLTF’s absence, G4s at the replication fork are bypassed by PrimPol-mediated repriming.

G4 stabilization can inhibit replication fork progression,^19–21,35,92^ and HLTF promotes fork reversal which restrains fork progression in response to replication stress-inducing reagents.^57^ Intriguingly, our data indicate that HLTF-KO cells have increased G4s and RNA-DNA hybrids, yet they exhibit fork progression rates similar to the parental cells.^47,57^ We thus wondered if HLTF loss allows cells to tolerate increased levels of these secondary structures. To address this question, we challenged cells with PDS and carried out a DNA fiber assay (Figure 7A). PDS treatment inhibited replication fork progression in the parental U2OS cells. In HLTF-KO cells, however, DNA synthesis continued at rates similar to untreated cells (Figure 7B). This suggests that HLTF slows fork progression upon G4 stabilization.

DNA synthesis is sustained in HLTF-deficient cells by PrimPol following treatment with HU.^57^ PrimPol can also bypass G4s formed at replication forks to prevent replicative helicase and polymerase uncoupling.^93^ Therefore, we asked whether PrimPol promotes fork progression following PDS treatment in HLTF’s absence. Indeed, fork progression was reduced dramatically following PDS challenge in PrimPol-HLTF double-knockout (dKO) cells (Figure 7B). We conclude that PrimPol promotes the bypass of PDS-stabilized G4s at replication forks in HLTF’s absence.

The knockdown of MSH2 elevates G4 and RNA-DNA hybrid levels in a manner that is enhanced when HLTF is also lost, and both structures slow replication fork progression and cause replication stress.^19,20,85,86,92,94^ Consistent with this idea, we found that MSH2 knockdown reduced fork progression in WT cells even in unchallenged conditions (Figure 7C). However, the knockdown of MSH2 in HLTF-KO cells did not slow fork progression. Furthermore, DNA synthesis was reduced in HLTF-PrimPol dKO cells (Figure 7C). Taken together, these data suggest that HLTF restrains fork progression upon increased formation of secondary DNA structures and that its loss promotes continued, PrimPol-dependent fork progression in this context. Hence, in the absence of HLTF, cells can continue DNA synthesis, effectively tolerating a variety of impediments to fork progression.

### HLTF protects cells from DNA damage and growth defects induced by G4 ligands

The ability of HLTF-deficient cells to replicate their DNA upon PDS challenge or in the absence of MSH2 led us to ask about the longer-term effects of elevated G4 levels on cells. The bypass of G4s with PrimPol might allow continued DNA synthesis but it could leave G4s unresolved, causing genome instability.

To determine how G4 formation and stabilization affect genome stability and growth of HLTF-deficient cells, we examined the formation of γH2AX foci, a marker for DNA damage. To minimize potentially confounding effects of telomeric damage, we assessed γH2AX foci in ALT-negative p53-deficient RPE1 cells. The knockdown of HLTF increased G4 formation in these cells in both G_1_ and G_2_/M phase, although MSH2 knockdown had little effect on G4s in these cells (Figure S7A). Interestingly, upon HLTF knockdown, we also observed an increase in γH2AX foci that was enhanced upon PDS treatment predominantly in S and G_2_/M phase cells (Figure 7D). Thus, although HLTF loss allows cells to continue DNA synthesis when G4s are stabilized, increased DNA damage is still observed.

Finally, we evaluated the impact of PDS on the proliferation of RPE1 cells, comparing it to the effects of HU and MMC treatments, to which HLTF-deficient cells exhibit resistance.^57^ Treatment with PDS slowed proliferation to a greater extent in HLTF-KOs relative to wild-type cells, whereas treatment with HU or MMC had the opposite effect (Figure S7B). We also found that the knockdown of MSH2 reduced proliferation upon increasing doses of PDS, although to a lesser extent than HLTF knockdown. The knockdown of both HLTF and MSH2 led to an even greater effect on proliferation (Figure 7E, F). Similar trends were observed after treatment with CX-5461, another G4-stabilizer (Figure S7C, D).^95^ Collectively, these data suggest that HLTF and MSH2 act in distinct pathways to prevent DNA damage and promote cell proliferation when G4s are stabilized.

## Discussion

Here, we describe a novel function for HLTF in maintaining genome stability, directly unfolding G4 structures and suppressing their accumulation throughout the cell cycle. In cells, HLTF requires its ATPase activity to suppress G4s, but not its ubiquitin ligase or HIRAN functions, whereas all three of these activities are essential for the HLTF-mediated replication stress response. Thus, the functions of HLTF in G4 resolution and the replication stress response are distinct (Figure 7G). Intriguingly, complete loss of HLTF facilitates PrimPol-dependent DNA synthesis, conferring tolerance to G4-induced replication stress. The consequence of this is increased DNA damage. Consistent with a role in preventing G4 accumulation, HLTF suppresses ALT activity and maintains telomere stability. Importantly, HLTF deficiency also sensitizes cells to G4 stabilization. This effect is enhanced by the loss of MSH2, which synergistically suppresses G4s. Notably then, HLTF silencing, which is often observed in cancers, may have a dual effect on cells, allowing the accumulation of mutagenic secondary DNA structures and permitting the replication fork to tolerate their presence.

### A function for HLTF in suppressing G4 accumulation

Several lines of evidence suggest that HLTF suppresses G4 structures in cells. First, G4s accumulate in the absence of HLTF. Second, HLTF is enriched at G4s genome-wide, with its greatest enrichment at sites where multiple G4s form. Third, there is an increase in RNA-DNA hybrids, which often co-occur with G4s, in HLTF-deficient cells. Although this increase was not observed in a previous study that reported no effect of HLTF knockdown on hybrid levels,^96^ the increase is modest, in a different cell line and was measured using a different approach for hybrid detection that may be more sensitive.^81^ Importantly, our genomic data further support the link between HLTF, RNA-DNA hybrids and G4s, as HLTF is particularly enriched at the small subset of hybrids (~15%) which coincide with G4s. Notably, the concurrent formation of G4s and RNA-DNA hybrids occurs at telomeres, where their deregulation increased ALT activity.^15^ Consistent with this, we observe an increase in ALT activity in HLTF-KO cells, and prior studies show HLTF localizes to telomeres in ALT-positive cells.^97^ Altogether, our data indicate that G4s, not RNA-DNA hybrids, drive HLTF’s localization to chromatin, and that stabilization of a G4 with a hybrid or its proximity to additional G4s further promotes this localization.

### A transcription-associated function for HLTF in the control of secondary structure accumulation

HLTF’s ability to suppress G4s is distinct from its known roles in DNA damage tolerance. HLTF requires its ATPase domain to suppress G4s, but not its HIRAN or ubiquitin ligase domains, which are needed for DNA damage tolerance in S phase. Indeed, G4 structures accumulate throughout the cell cycle upon HLTF loss. Also consistent with a role outside of S phase, more than half of HLTF’s chromatin binding is transcription dependent (Figure 1H). Since transcription facilitates the formation of G4s,^9^ the reduction in HLTF chromatin binding following transcription inhibition may reflect reduced formation of these structures. Intriguingly, HLTF was originally identified as a transcription factor because it binds certain promoter motifs and positively regulates transcription in reporter assays.^44,45^ Although the mechanism underlying this observation has not been investigated, our findings support a role for HLTF in transcription. Interestingly, SMARCAL1 has been shown to resolve RNA-DNA hybrids *in vitro*^96^ and to modulate transcription by controlling chromatin accessibility.^98^ If and how HLTF also regulates transcription, and whether it is linked to its regulation of secondary DNA structures, warrants further study.

### Proposed mechanism for HLTF-dependent regulation of G4s

Our data, taken together with previously published biochemical studies, suggest a new biochemical mechanism for the resolution of G4s by HLTF. We found that HLTF does not specifically bind to or resolve G4 structures in ssDNA. However, we observed ATP-dependent G4 resolution in the context of dsDNA, when the two strands could be reannealed. Prior studies have shown that HLTF translocates on dsDNA and can move through and resolve triplex forming oligonucleotides when duplex DNA is intact.^53^ Hence, we propose that HLTF travels on dsDNA until it encounters a G4. Its translocase activity then destabilizes this structure, effectively unfolding it, and simultaneously promotes reannealing of the structure-forming ssDNA with its complementary strand to ultimately form duplex DNA. This biochemical process is similar to HLTF’s activity during fork reversal, where it reanneals the parental strands while simultaneously displacing and annealing the nascent strands. During fork reversal, but not G4 unfolding, the 3’ DNA-end binding activity of the HIRAN domain is also needed to facilitate remodeling of the nascent strands. This is consistent with our finding that the HIRAN domain is not needed for G4 unfolding and the lack of a 3’ DNA end in this structure.

This mechanism for G4 resolution raises the possibility that HLTF may suppress the formation of other secondary DNA structures, removing them in a similar manner. Indeed, HLTF can reduce the expansion of some trinucleotide repeats^99,100^ that form DNA hairpins or H-DNA, and their expansion is linked to the secondary structure-forming properties of the sequence. However, these studies did not identify the HLTF domain(s) required for expansion or clearly distinguish between potential S phase or non-S phase functions of HLTF. As the analysis of many secondary structures in cells remains a challenge, the role of HLTF in regulating other structures will require further study. Finally, our work raises the possibility that other dsDNA translocases that act in a similar manner as reannealing helicases, such as SMARCAL1 and ZRANB3, may also have the ability to resolve secondary structures.

### The synergy between HLTF and MSH2 during G4 regulation

Several lines of evidence suggest that HLTF and MSH2 have complementary roles in regulating G4s. First, the chromatin binding of each protein increases when the other is lost, suggesting that each protein may compensate for the other in suppressing G4s, RNA-DNA hybrids and potentially other structures. Second, a synergistic increase in G4s was observed when both HLTF and MSH2 were absent. Third, there was a further increase in cellular sensitivity to PDS when both proteins were lost. Finally, HLTF and MSH2 act synergistically to suppress ALT activity.

It seems likely HLTF and MSH2 use different mechanisms to resolve G4s. HLTF does not specifically bind to G4s but can resolve them *in vitro*. By contrast, the MutSα and β complexes can directly bind to G4s and MutSβ can destabilize them as well.^32,33^ Hence MSH2, as part of the MutSβ complex, may suppress G4s through this binding and destabilization. Alternatively resolution may be indirect, as the MutS complexes interact with multiple G4 resolving helicases.^101,102^ Interestingly, MutSβ has been shown to recruit FANCJ through MLH1, ^34,103^ and similar to MSH2 loss, FANCJ loss also leads to increased HLTF chromatin binding.^104^ Although our findings support complementary roles for HLTF and MSH2, they do not rule out the possibility that HLTF and MSH2 may act cooperatively on some G4s. For example, MSH2 could help to melt some G4s, while HLTF promotes reannealing of the melted G4 DNA with its complementary DNA strand.

### HLTF and the tolerance of G4 structures

We previously found that HLTF loss promotes resistance to replication stress induced by several compounds, including HU, ATR inhibitors and MMC. In response to HU and MMC, we also showed that replication forks continue DNA synthesis when HLTF is depleted. This correlation led us to speculate that unrestrained fork progression may underlie the observed replication stress resistance.^57^ In response to G4 ligands, however, this is not the case. Although DNA synthesis continues in HLTF-KO cells using PrimPol, the absence of HLTF sensitizes cells to G4 ligands, resulting in increased DNA damage and reduced proliferation. Therefore, despite the ability of cells to continue DNA replication in the presence of PDS, the stabilized G4s are still problematic and can cause DNA damage. This damage could result from the inability to resolve G4s in post-replicative gaps and subsequent processing of these structures and is consistent with the higher level of γH2AX observed in S and G_2_/M cells. Thus, our new data indicate that unrestrained fork progression is not sufficient for replication stress resistance in HLTF-deficient cells.

### Limitations of the study

In our analysis of cellular G4 levels, we used G4 structure-specific antibodies. Although the different antibodies produced consistent results, we observed minor differences in the signal. This could reflect the ability of different antibodies to bind to G4 structures of distinct topologies or to recognize G4s in RNA.^6^ It is possible that HLTF can resolve G4s at the fork using its fork reversal activity, but that our assay is not sensitive enough to detect the small fraction of such events that occur compared to the total number of G4s. Additionally, we analyzed HLTF’s interaction with G4 sites in the genome that were identified using a single G4 antibody. Although this antibody has been used in both G4 ChIP-seq and CUT&Tag in the same cell type, and the sites identified using both approaches significantly overlap,^75^ some G4s may have escaped detection resulting in an underestimation of HLTF’s overlap with G4s. We also found HLTF’s enrichment is increased at G4s stabilized by RNA-DNA hybrids, identifying these genomic regions by intersecting peaks identified by DRIP-seq and G4 CUT&Tag. Since both RNA-DNA hybrids and G4s were detected using population-based approaches, these structures may not form concurrently at the intersected sites. Finally, further biochemical studies are needed to explore HLTF’s ability to bind or resolve other types of G4s.

### Conclusions

In summary, our findings reveal an unexpected function for HLTF in the regulation of secondary DNA structures, maintaining G4s homeostasis throughout the cell cycle and in a manner that is linked to transcription. Intriguingly, HLTF’s functions in fork reversal and damage tolerance may synergize with its G4 resolution function to prevent the mutagenic and potentially toxic effects of these structures (Figure 7G). HLTF-dependent fork slowing may prevent alternative modes of replication and promote the resolution of G4s and other DNA secondary structures. Hence, HLTF loss elevates the formation of these replication stress-inducing structures yet allows their bypass and eventual resolution by potentially mutagenic pathways. In the context of cancer, HLTF silencing could thus both elevate replication stress resulting from G4s and increase the tolerance to that stress, driving mutagenesis. In the long term, this may pose an important therapeutic opportunity for the treatment of HLTF-deficient cells with G4 stabilizing drugs.

## Star Methods

### Cell Culture

U2OS (osteosarcoma; human female origin, ATCC #HTB-96) cells were maintained in DMEM (Life Technologies) supplemented with 10% FBS, 2 mM L-glutamine, and 100 U/mL penicillin/streptomycin. MCF10A (mammary gland epithelial; human female origin, ATCC #CRL-10317) cells were maintained in DMEM/F12 (Life Technologies) supplemented with 5% horse serum, 100 U/mL penicillin/streptomycin, 0.5 μg/mL hydrocortisone, 10 μg/mL insulin, 20 ng/mL EGF, and 100 ng/mL Cholera Toxin. p53 inactivated, Cas9 expressing hTERT-RPE1 (immortalized; retinal pigment epithelium; human female origin, ATCC #CRL-4000) cells described as RPE1 in the manuscript were received as a kind gift from Dr. Daniel Durocher and maintained in DMEM/F12 (Life Technologies) supplemented with 10% FBS, 100 U/mL penicillin/streptomycin. All cell lines were tested for the absence of mycoplasma contamination and cultured in a humidified incubator with 5% CO_2_ at 37°C.

### Construction of Cas9 expressing cells

Lentivirus encoding a construct that constitutively expresses S. pyogenes Cas9 was purchased (Addgene 52962-LV) and used to infect U2OS and MCF10A cells in the presence of polybrene (1 μg/mL, Millipore) overnight. 48 h post-infection, 10 μg/mL (for U2OS cells) and 5 μg/mL (for MCF10A cells) blasticidin was added to the media to select for the infected cells. Cas9 expression in the resistant cells was verified by western blotting.

### Gene expression knockdown using siRNA or sgRNA

An siRNAs smart pool for MSH2 was purchased from Dharmacon and transfected using Dharmafect 1 (Horizon) transfection reagent according to the manufacturer’s directions. siRNA against GL3 luciferase was used as a negative control. sgRNA against HLTF and MSH2 (Thermo Fisher Scientific) were transfected into Cas9 expressing U2OS, RPE1 or MCF10A cells using Lipofectamine RNAiMAX (Thermo Fisher Scientific) according to the manufacturer’s directions. A non-targeting sgRNA was used as a negative control. siRNA transfected cells were assayed 72 h post-transfection to test gene expression knockdown by western blotting, or used for other assays. sgRNA transfected RPE1 cells were assayed 72 h post-transfection, U2OS cells were assayed 96 h post-transfection to test gene expression knockdown by western blotting or immunofluorescent imaging, or used for other assays.

### Construction of rescue cell lines

HLTF’s cDNA on the pcDNA3.1(+) backbone^57^ was mutated using Q5 Site-Directed Mutagenesis kit (NEB) to generate the C760S and R890Q mutant, then cloned into pCW57.1 using HiFi assembly kit (NEB). All the plasmids were sequenced and verified. pCW57.1-HLTF vectors were packaged into lentivirus particles using the 2nd generation lentiviral packaging system (pMD2.G & psPAX2) in HEK293T cells using TransIT®-LT1 Transfection Reagent (Mirus). Virus-containing media was harvested 24 & 48h post-transfection and filtered through 0.45 μm PES membrane syringe filter to eliminate packaging cells. Lentivirus particles were further concentrated using Lenti-X Concentrator (Clontech) according to the manufacturer’s instructions. U2OS HLTF-KO cells were infected with the purified lentivirus particles in the presence of polybrene (1 μg/mL, Millipore) overnight. 48h post-infection, 1 μg/mL puromycin was added to the media to start the selection of infected cells. The resistant cells were clonally isolated using cloning cylinders. Resistant clones were further selected after doxycycline (dox) induction (500 ng/mL) for 24h and HLTF expressing clones were identified by immunofluorescence staining and also verified by western blotting. Two clones for each genotype were analyzed separately and the results were later pooled and presented.

Although designed as a dox-inducible expression system, we observed that the expression of the WT, R71E and C760S mutant was similar to HLTF’s endogenous level even in the absence of dox.^57^ In experiments involving HLTF-KO cells expressing the WT and these mutants, dox was omitted. We failed to detect the expression of the R890Q mutant in HLTF-KO cells unless dox was used to induce protein expression. Therefore, in experiments evaluating HLTF-KO cells expressing the R890Q mutant, cells were either mock or treated with dox (500 ng/mL, 24h) to induce R890Q expression before assessing other phenotypes.

### iPOND-SILAC Mass Spectroscopy and Data Analysis

iPOND was performed as described previously.^46^ SILAC-labeled HEK293T cells were pulsed with 10 µM EdU with the presence of 50 µM HU for 13 min for the WT, and 10 min for the HLTF-KO cells. After labeling, roughly 6x10^8^ asynchronous cells for each sample were fixed in 1% formaldehyde/PBS for 20 min at room temperature (RT), quenched using 0.125 M final concentration of glycine, and washed three times in PBS. Cells were scraped off the tissue culture dishes and the cell pellets were frozen at −80°C. Pellets were then resuspended in 0.25% Triton-X/PBS to permeabilize. Pellets were washed once with 0.5% BSA/PBS and once with PBS prior to the click reaction. An equal number of light and heavy SILAC-labeled cells were mixed and resuspended in a homemade click reaction buffer (10 μM biotin-PEG-azide, 2 mM CuSO_4_, 10 mM sodium L-ascorbate in PBS, freshly prepared) and incubated for 1h at RT. After cell lysis by sonication, biotinylated chromatins were captured using streptavidin-coupled C1 magnetic beads for 1h at RT, then washed with lysis buffer (1% SDS in 50 mM Tris-HCl pH 8.0), low salt buffer (1% Triton X-100, 20 mM Tris-HCl pH 8.0, 2 mM EDTA, 150 mM NaCl), high salt buffer (1% Triton X-100, 20 mM Tris-HCl pH 8.0, 2 mM EDTA, 500 mM NaCl), lithium chloride wash buffer (100 mM Tris-HCl pH 8.0, 500 mM LiCl, 1% Igepal), and twice in lysis buffer. Captured proteins were eluted in SDS sample buffer by incubating for 30 min at 95°C.

iPOND samples were then processed and analyzed using mass spectrometry as described previously.^48^ For SILAC protein ratios, a minimum of two unique peptides and one or more ratio counts were required for protein group inclusion in the analysis. SILAC protein ratios for all datasets were analyzed within the Perseus software.^105^ Only proteins identified in both datasets were included in the statistical analysis in Perseus using a two-tailed t-test with a p-value set at 0.05.

### Immunofluorescent staining and imaging

Cells were seeded in 96 well optical plate and incubated overnight. The next day, 10 μM EdU diluted in cell culture media was added to label S phase cells for 30 min. After labeling, cells were pre-extracted with ice-cold CSK100 (100 mM NaCl, 300 mM sucrose, 3 mM MgCl_2_, 10 mM MOPS & 0.5% Triton X-100) buffer at 4°C for 5 min, then immediately fixed with 4% PFA/PBS for 20 min. After PBS washes, cells were permeabilized with 0.5% Triton X-100 in PBS for 5 min. A homemade click reaction buffer (10 μM biotin-PEG-azide, 2 mM CuSO_4_, 10 mM sodium L-ascorbate in PBS, freshly prepared) was added to the cells for 30 min, followed by blocking in 3% BSA/PBS for 20 min at RT. The primary antibodies were diluted in 3% BSA/PBS and incubated overnight at 4°C: rabbit anti-G4 (clone 1H6, Absolute Antibody ab00389-23.0, 1:500), goat anti-G4 (clone 1H6, Absolute Antibody ab00389-24.1, 1:500), anti-G4 (clone BG4, Sigma-Aldrich MABE917, 1:500), rabbit anti-GFP (Abcam ab290, 1:1000), rabbit anti-GFP (Thermo Fisher Scientific A-11122, 1:1000), rabbit anti-HLTF (Abcam ab183042, 1:1000), mouse anti-MSH2 (Abcam ab52266, 1:1000), mouse anti-RNA polymerase II CTD repeat YSPTSPS (phospho S2) (Abcam ab24758, 1:500), rabbit anti-RPA34 (Abcam ab97594, 1:1000). For G4 detection using BG4 antibody, rabbit anti-DYKDDDK (Cell Signalling Technology 14793S, 1:500) was diluted in 3% BSA/PBS and incubated for 1 h at RT. After primary antibody incubation, cells were washed 3x with PBS. Secondary antibodies (diluted 1:500) and DAPI (2μg/mL) were diluted in 3% BSA/PBS and incubated for 1 h at RT. Cells were washed 3x with PBS and then submerged in PBS during QIBC image acquisition.

### RNA-DNA hybrid detection by GFP-dRNH1 using fluorescent imaging

RNA-DNA hybrid detection by GFP-dRNH1 was performed as described previously.^81^ Cells were seeded in 96 well optical plate and incubated overnight. Next day, 10 μM EdU diluted in cell culture media was added to the cells to label S phase cells for 30 min. After labeling, cells were fixed with ice-cold methanol for 10 min at −20°C. RNase H diluted (1:100) in 1× RNase H buffer (New England Biolabs) was added to the cells and incubated for 2 h at 37°C. Mock-treated cells were incubated in parallel in the same buffers but without RNase H added. Following enzyme incubations, cells were washed twice in PBST (0.1% Tween 20 in PBS), then once in PBS for 5 min each. A homemade click reaction buffer (10 μM biotin-PEG-azide, 2 mM CuSO_4_, 10 mM sodium L-ascorbate in PBS, freshly prepared) was added to the cells for 30 min to detect EdU incorporated S phase cells, followed by blocking in 3% BSA/PBS for 30 min at RT. GFP-dRNH1 (0.188 mg/ml stock) diluted in 3% BSA/PBS (1:2000) was added to the cells and incubated overnight at 4°C. Then, cells were washed twice in PBST and once in PBS for 5 min each. Streptavidin-conjugated Alexa 647 (diluted 1:500) and DAPI (2 μg/mL) were diluted in 3% BSA/PBS and incubated for 1 h at RT. Cells were washed 3x with PBS and then submerged in PBS during QIBC image acquisition.

### Quantitative Image-Based Cytometry (QIBC)

Images were acquired in an unbiased fashion with the Molecular Devices ImageXpress Micro automated inverted epifluorescence microscope. Acquisition times for different channels were adjusted to obtain images in non-saturating conditions for all the treatments analyzed. After acquisition, the images were analyzed with automated MetaXpress image analysis software. At least 2000 cells were analyzed per condition, and each experiment was repeated at least 3 times. DAPI signal was used for generating a mask that identified each individual nucleus as an individual object. Large RPA foci were identified after applying a top hat filter to the RPA staining channel, then a mask was generated after filtering. These masks were then applied to quantify pixel intensities in the different channels for each individual cell/object. After quantification, the quantified values for each cell or foci (mean and total intensities, area, perimeter) were extracted and exported to the proprietary Spotfire software. Spotfire was used to visualize key features of individual nuclei for thousands of cells and quantify immunofluorescence values at the single cell level. Spotfire filtered data was then used to generate plots using GraphPad Prism [version 10.0.1 (218) for Windows 64-bit, GraphPad Software, La Jolla California USA, https://www.graphpad.com] software.

Whenever cells were labeled with EdU, total DNA content and mean EdU intensities of individual cells were used to determine cell cycle phases. Cells with positive EdU staining were considered as S phase cells. Total DNA content of the EdU negative cells were used to distinguish G_1_ (2N DNA content) from G_2_/M (4N DNA content) phase.

### ALT-associated PML body detection

Cells were seeded in 24 well optical plate and incubated overnight. The next day, cells were pre-extracted with ice-cold CSK100 (100 mM NaCl, 300 mM sucrose, 3 mM MgCl_2_, 10 mM MOPS & 0.5% Triton X-100) buffer at 4°C for 5 min, then immediately fixed with 4% PFA/PBS for 20 min. After PBS washes, cells were permeabilized with 0.5% Triton X-100 in PBS for 5 min and blocked in blocking solution (0.1% BSA, 3% goat serum, 0.1% Triton X-100,1 mM EDTA, pH 8.0 in PBS) for 30 min at RT. The primary antibodies were diluted in 3% BSA/PBS and incubated overnight at 4°C: rabbit anti-RPA34 (Abcam ab97594, 1:1000), mouse anti-PML (Santa Cruz Biotechnology sc-966, 1:200). After the primary antibody incubation, cells were washed 3x with PBS. Secondary antibodies (diluted 1:500) were diluted in 3% BSA/PBS and incubated for 1 h at RT. A second fix was performed with 2% PFA/PBS for 10 min at RT. After PBS washes, samples were then serial dehydrated with 70%, 95% and 100% ethanol for 5 min each and let air dry. Biotin-tagged TelC probe (PNA Bio F2001) was diluted (1:200) in hybridization solution [70% formamide, 1 mg/mL Blocking Reagent (Roche 11096176001), 10 mM Tris-HCl pH 7.2, in ddH_2_O]. Samples and the diluted probe were both incubated at 80°C for 5 min, before the probe was added to the sample and incubated at 80°C for another 10 min, followed by incubation at RT for 2 h. Samples were then washed twice with washing solution (70% formamide, 10 mM Tris-HCl pH 7.2, in ddH2O) and twice with PBS, 3 min each wash. Streptavidin conjugated Alexa 647 (diluted 1:500) and DAPI (2 μg/mL) were diluted in 3% BSA/PBS and incubated for 1 h at RT. Cells were washed 3x with PBS and then coverslips were mounted using ProLong Glass antifade (Thermo Fisher Scientific P36984). Random views were selected and imaged using a Zeiss OBSERVER.Z1 INVERTED microscope and a Plan-APO 40x/1.4 Oil DIC (UV) VIS-IR objective. Fluorescent images were acquired using an Axiocam 506 mono camera (conversion = 0.1135) connected to the microscope. Images were analyzed using ImageJ Fiji^106^ to count the number of PML and telomere FISH foci that colocalize per in each cell.

### In situ single-stranded telomeric C-strand (ssTelo-C) detection

Cells were seeded in a 24 well optical plate and incubated overnight. The next day, cells were fixed with 4% PFA/PBS for 20 min. ssTelo-C was stained as described previously.^91^ Briefly, samples were incubated with 500 μg/mL RNase A (Thermo Fisher Scientific EN0531) diluted in blocking solution (0.1% BSA, 3% goat serum, 0.1% Triton X-100, 1 mM EDTA, pH 8.0 in PBS) for 1h at 37°C. Samples were then washed once with PBS, then serial dehydrated with 70%, 95% and 100% ethanol for 5 min each and let air dry. FAM-tagged TelG probe (PNA Bio F1005) was diluted (1:100) in hybridization solution [70% formamide, 1 mg/mL Blocking Reagent (Roche 11096176001), 10 mM Tris-HCl pH 7.2, in ddH_2_O] and added to the samples to incubate for 2 h at RT. Samples were washed twice with washing solution (70% formamide, 10 mM Tris-HCl pH 7.2, in ddH2O) and twice with PBS, 3 min each wash. Nuclei were counterstained with DAPI (2 μg/mL) diluted in PBS and incubated for 1 h at RT. Cells were washed 3x with PBS and then coverslips were mounted using ProLong Glass antifade (Thermo Fisher Scientific P36984). Random views were selected and imaged using a Zeiss OBSERVER.Z1 INVERTED microscope and a Plan-APO 40x/1.4 Oil DIC (UV) VIS-IR objective. Fluorescent images were acquired using an Axiocam 506 mono camera (conversion = 0.1135) connected to the microscope. Images were analyzed using ImageJ2 Fiji to count the number of single-stranded TelC foci per cell.

### ChIP-seq with spike-in normalization (ChIP-Rx)

For each ChIP-Rx sequencing experiment, 6x10^7^ cells per immunoprecipitation condition were fixed with 1% formaldehyde (Fisher Chemical, F79-500) for 5 min at RT. Fixation was stopped by adding 125 mM glycine for 5 min. Cells were harvested in ice-cold PBS containing protease (Sigma-Aldrich, 11836170001) and phosphatase inhibitors (Roche, 4906837001). All further used buffers also contained these inhibitors. As exogenous control (spike-in), murine ESC cells expressing MPP8-GFP (gift from Wysocka Lab^107^) were added at a 1:10 ratio during cell lysis. Cell lysis was carried out for 20 min in lysis buffer I (5 mM PIPES pH 8.0, 85 mM KCl, 0.5% NP-40 in nuclease-free water) and nuclei were collected by centrifugation at 500 g for 20 min at 4 °C. Crosslinked chromatin was prepared in lysis buffer II (10 mM Tris-HCl pH 7.5, 150 mM NaCl, 1 mM EDTA, 1% NP-40, 1% sodium deoxycholate, 0.1% SDS in nuclease-free water) and fragmented by using the Covaris Focused Ultrasonicator E220 for 20 min (10% duty factor, 140 PIP, 200 CPB) per mL lysate. Fragment size of 150-300 bp was validated by agarose gel electrophoresis. Chromatin was centrifuged for 20 min at 14,000 rpm at 4 °C before IP. For each IP reaction, 100 μl Dynabeads Protein A and Protein G (Thermo Fisher Scientific, 10002D and 10004D) were pre-incubated overnight with rotation in the presence of 5 mg/ml BSA/PBS (Thermo Fisher Scientific, BP1600-100) and 15 μg antibody GFP (Abcam, ab290). Chromatin was added to the beads, and IP was performed for at least 6 h at 4 °C with rotation. Beads were washed three times each with washing buffer I (20 mM Tris-HCl pH 8.1, 150 mM NaCl, 2 mM EDTA, 1% Triton X-100, 0.1% SDS in nuclease-free water), washing buffer II (20 mM Tris-HCl pH 8.1, 500 mM NaCl, 2 mM EDTA, 1% Triton X-100, 0.1% SDS in nuclease-free water), washing buffer III (10 mM Tris-HCl pH 8.1, 250 mM LiCl, 1 mM EDTA, 1% NP-40, 1% sodium deoxycholate in nuclease-free water), and once with TE buffer (Invitrogen). Chromatin was eluted twice by incubating with 150 mL elution buffer (100 mM NaHCO3, 1% SDS) for 15 min with rotation at RT. Input samples and eluted samples were de-crosslinked overnight at 65 °C. Protein and RNA were digested with proteinase K (Thermo Scientific, 25530049) and RNase A (Thermo Scientific, EN0531), respectively. DNA was isolated by phenol-chloroform extraction and ethanol precipitation and analyzed by qPCR using Light Cycler Real-Time PCR System (Roche) and iTaq Universal SYBR Green Supermix SYBR Green Master Mix (BioRad, 1725122) to perform ChIP qPCR or before library preparation followed by high throughput sequencing on a Novaseq 6000. See Key Resource Table for qPCR primer details.

For ChIP-Rx sequencing, DNA was quantified using the Quant-iT™ dsDNA HS Assay Kit (Invitrogen, Q32851). DNA library was prepared using the NEBnext Ultra II DNA Library Prep Kit (New England Biolabs, E7645) and NEBNext Multiplex Oligos for Illumina (NEB, E7600) following the manufacturer’s instructions. Cycle number was determined by qPCR (Thermo Scientific, S11494). Quality and Quantity of the library were assessed on the QUBIT using the Quant-iT™ dsDNA HS Assay Kit, the 2100 Bioanalyzer (Agilent) using the High Sensitivity DNA Assay and NEBNext Library Quant Kit for Illumina (NEB, E7630). Finally, libraries were subjected to cluster generation and base calling for 150 cycles paired-end on the Illumina Novaseq 6000 platform.

### Bioinformatics analysis

For HLTF ChIP-seq samples, FASTA files of human (hg38) and mouse (mm39) reference genome were downloaded from RefSeq (https://www.ncbi.nlm.nih.gov/datasets/genome/). Both FASTA files were used in bowtie2-build to generate an indexed “hybrid” reference genome. Raw FASTQ reads from HLTF ChIP-seq were aligned to the hybrid reference genome using bowtie2^108^ (2.2.5) –local mode using paired inputs with default parameters. Duplicates were identified using Picard (2.27.5) MarkDuplicates. Samtools^109^ (1.16.1) was used to filtered the reads: reads aligned to the chromosomes were kept; unmapped, pairs with only one mate aligned, duplicates and reads aligned to the ENCODE blacklist regions were excluded.^110^ After filtering, the reads are parsed into human versus mouse aligned reads. The total number of mouse aligned reads for each sample were taken to calculate the spike-in normalization factors as previously described.^74^ Deeptools^111^ (3.5.1) bamCoverage was used to generate bigwig files with the signals scaled using the spike-in normalization factor. The resulting bigwig files were used in deeptools computeMatrix, followed by plotHeatmap and plotProfiles to generate the heatmaps and aggregate plots respectively. For G4 ChIP-seq (GSE162299) and MSH2 ChIP-seq (GSE205043) in the mouse embryonic stem cells (mECSs, E14), raw reads in FASTQ files were downloaded and aligned to human (hg38) or mouse (mm10) reference genome using bowtie2 (2.2.5) –local mode with default parameters. The aligned reads were deduplicated and filtered as mentioned above. Deeptools (3.5.1) bamCoverage was used to generate bigwig files with the signals normalized to 1x genome size, using the reads per genome content (RPGC) method. Bed files of the G4 CUT&Tag peaks (GSE181373), DRIP peaks (GSE115957) identified in the U2OS cells and *in vitro* G4 forming motifs in the human genome under PDS^+^, K^+^ condition (GSE110582) were downloaded and used to calculate HLTF ChIP-seq signal enrichment. Bed files of the G4 CUT&Tag-seq (GSE70189), DRIP-seq (GSE173103) in the mESCs were downloaded and used to calculate MSH2 ChIP-seq signal enrichment. Liftover (https://genome.ucsc.edu/cgi-bin/hgLiftOver) was used to convert genome coordinates to those in the hg38 assemblies. MACS2^112^ (2.2.7.1) was used to identify HLTF ChIP-seq peaks with default settings using FDR=0.05 as cutoff. The peaks were further filtered based on the average ChIP-seq signal intensity within the peak regions following ENCODE guidelines ^113^: at least a 2-fold signal enrichment in the control versus HLTF knockdown ChIP-seq sample was used as cutoff. Bedtools^114^ (2.30.0) was used to intersect G4 CUT&Tag, G4 motifs, DRIP and HLTF ChIP-seq peaks. HOMER^115^ (4.11) was used to analyze the basic genome ontology of G4 CUT&Tag and HLTF ChIP-seq peaks.

### DNA fiber analysis with triple nucleotide labeling

Cells were seeded in 24 well plate and incubated overnight. Unless otherwise stated in the figure legends, on the next day, cells were labeled consecutively with 50 μM IdU, 100 μM EdU and 200 μM CldU for 30, 30 and 20 min each. Warm PBS was applied to wash cells between each labeling. Drug or mock treatment was performed during the EdU labeling. After the CldU labeling, cells were trypsinized and harvested. Chromatin fibers were spread on glass slides and then fixed with freshly prepared fixative (75% methanol, 25% acetic acid) for 10 min at RT. To detect nucleotide analogue labeled fibers, slides were denatured with 2.5N HCl for 30 min at RT. A homemade click reaction buffer (100 μM biotin-PEG-azide, 2 mM CuSO_4_, 10 mM sodium L-ascorbate in PBST, freshly prepared) was added to the slides and incubated for 30 min at RT. Primary antibodies were diluted in 3% BSA/PBST (0.1% Triton X-100 in PBS) and incubated with the samples at 37°C for 1 h: mouse anti-BrdU (BD Biosciences 347580, 1:75, for IdU detection), rat anti-BrdU (Abcam ab6326, 1:100, for CldU detection), rabbit anti-biotin (Cell Signaling Technology 5597, 1:100, for biotinylated EdU detection after click reaction). PBST was used to wash the slides 3 times. Secondary antibodies diluted in 3% BSA/PBST were incubated with the samples at 37°C for 1 h: goat anti-rat IgG (H+L) (Thermo Fisher Scientific A11006, 1:100), goat anti-rabbit IgG (H+L) (Thermo Fisher Scientific A21244, 1:100), goat anti-mouse IgG1 (Thermo Fisher Scientific A21124, 1:100). Samples were then washed with PBST and let air dry, before mounted with Fluoroshield (Sigma-Aldrich F6182) using 1.5N coverglass. Random views were selected and imaged using a Zeiss OBSERVER.Z1 INVERTED microscope and a Plan-APO 40x/1.4 Oil DIC (UV) VIS-IR objective. Fluorescent images were acquired using an Axiocam 506 mono camera (conversion = 0.1135) connected to the microscope. To evaluate the replication fork progression rate, EdU tract lengths were measured only when preceded by IdU labeling and followed by CldU labeling. For quantification, at least 2 slides per sample were prepared for each experimental repeat. To avoid bias, after immunodetection, each pair of slides was blinded, and we randomly selected at least 5 fields of view from each slide and acquired images. Hence, for each experiment, we acquired at least 10 images for each sample. DNA fiber length was measured using an ImageJ plug-in. We randomly scored a similar number of fibers (~15) from each image. Measurements from at least 3 repeats were pooled, resulting in at least 200 replication tracts per sample.

### Protein purification

UBC13/MMS2 were expressed and purified as described previously.^116^ Human HLTF proteins used in ATPase, fork regression, ubiquitin ligase, binding and unwinding of G4 in ssDNA assays were expressed and purified as described previously.^56^ UBA1 (ab271808) and ubiquitin (ab269109) were purchased from Abcam. HLTF WT and HIRAN (N90A, N91A, or NANA) mutant proteins used in the dsDNA G4 unfolding assay were expressed and purified as described previously.^117^

### ATPase assay

The ATP hydrolysis rate of HLTF was determined using a NADH-coupled enzymatic assay.^118^ Briefly, the reactions were carried out in solution containing 20 mM Tris-HCl pH 7.76, 2 mM MgCl_2_, 10 mM KCl, 1 mM DTT, 2 mM ATP, 3 mM phosphoenolpyruvate, 0.044 mM NADH, 12 U pyruvate kinase, and 17 U lactate dehydrogenase, supplemented with 100 nM HLTF, and 300 nM fork DNA (assembled from oligos 40, F20.40, LEAD20.20, and LAG20.20; see Key Resource Table). The A340 was monitored for 60 min (1 min intervals) at 37°C in a 96-well plate (Corning) using a Synergy H1 Hybrid Reader (Biotek). Data were analyzed using GraphPad Prism and rates were corrected for background NADH decomposition from a no-enzyme control.

### Fork regression assay

Fork regression was performed as previously described.^56^ Reactions containing 5 nM HLTF and 1 nM ^32^P-labeled fork DNA assembled from oligos 48, 50, 52, and 53 (see Key Resource Table) were incubated at 37°C in reaction buffer (20 mM Tris-HCl pH 7.76, 2 mM MgCl_2_, 10 mM KCl, 1 mM DTT, 2 mM ATP, 100 µg/mL BSA).

### Ubiquitin ligase assay

DNA oligonucleotides Bio-31 and Bio-75 (see Key Resource Table) were annealed in annealing buffer (10 mM Tris pH 7.5, 50 mM NaCl, 1 mM EDTA) to create a duplex with a 5’-ssDNA overhang. Reactions were carried out in ubiquitylation buffer (40 mM Tris pH 7.76, 8 mM MgCl_2_, 10% glycerol, 50 mM NaCl) and contained 0.1 μM UBA1, 40 μM ubiquitin, 0.2 μM UBC13/MMS2, 0.2 μM HLTF, 0.05 μM Bio-31/Bio-75 DNA, and 0.5 mM ATP. Reactions were incubated at 30°C for 15 minutes and stopped by the addition of 2X Laemmli buffer followed by incubation at 70°C for 5 minutes. Samples were analyzed by western blot using anti-ubiquitin antibody (P4D1, Cytoskeleton AUB01-HRP, 1:1000) and visualized with Clarity Western ECL Substrate (BioRad).

### Protein binding to G4 in ssDNA

10 nM ssDNA was incubated in a buffer containing 40 mM Tris-HCl pH 8.0, 50 mM KCl, 5 mM MgCl_2_, 2 mM AMP-PNP, 1 mM TCEP and 0.1 mg/mL BSA. Two types of ssDNA were used: “Tel 8” ssDNA that contains 8 telomeric repeats and a control “No G4” ssDNA that contains equal number of scrambled telomeric repeats (see Key Resource Table). Purified HLTF protein was added to a final concentration of 0-500 nM in a final reaction volume of 10 μL. After incubation at 37°C for 10 min, glycerol was added to a final concentration of 10%. Electrophoresis was performed using a 0.7% agarose TBE gel at 70 volts for 1 h at 4°C. Gels were imaged on a ChemiDoc fluorescent imager. The intensities of the bound vs. unbound bands were quantified using ImageJ.

### ssDNA G4 unfolding assay

The ssDNA G4 unwinding assay was modified from a previous report.^119^ Briefly, 2 nM of Tel 8 ssDNA was incubated at 4°C in a buffer containing 40 mM Tris-HCl pH8.0, 50 mM KCl, 5 mM MgCl_2_, 2 mM ATP, 1 mM TCEP and 0.1 mg/ml BSA. For Phen-DC3 G4 stabilization experiments Phen-DC3 was added to a final concentration of 50 nM. 50 nM of HLTF or yeast Pif1^120^ and 5 nM G4 probe (see Key Resource Table) were added in a final reaction volume of 120 μL to initiate G4 unwinding. The reaction was incubated at room temperature. At 0, 1, 2, 5 and 10 min after the reaction was initiated, 20 μL of the reaction mixture was taken and combined with 6x Stop Solution (60 mM EDTA pH 8.0, 40% Glycerol, 0.6% SDS, 0.5 mg/ml Proteinase K) and kept at 4 °C. Samples were analyzed by electrophoresis in a native 10% polyacrylamide TBE Gel run in 1 x TBE buffer at 100 volts for 2 h at 4 °C. Both the gel and the running buffer contain 50 mM KCl. Gels were imaged on a ChemiDoc fluorescent imager. The intensities of the folded vs. unfolded bands were quantified using ImageJ.

### DNA substrate preparation for the dsDNA G4 unfolding assay

The pUC19-Random inverted repeats vector (pUC19-RIR, or No G4-DNA)^73^ was modified as follows: ssDNA containing a CEB25 minisatellite G4-forming sequence^121^ was modified to include an EcoRI restriction site, then annealed to its complementary ssDNA and inserted between the XbaI and SacI site on pUC19-RIR to create pUC19-G4 (or G4-DNA, see Key Resource Table). pUC19-G4 was linearized by ScaI-HF (New England Biolabs) and distributed into PCR tubes to include 1.5 μg of DNA, 3 μM pyridostatin (Sigma, SML2690), 50 mM KCl, 10 mM Tris-HCl pH 8.0, 1 mM MgCl_2_, and 100-fold excess of a complementary RNA in molecules (see Key Resources Table), in a total volume of 30 μL. The reaction mix was incubated at 95°C for 5 min followed by slow cooling (0.01 °C/sec). RNaseH (2.5 U, New England Biolabs) was used to digest RNA at 37°C for 30 min, followed by heat inactivation. The solutions were pooled and used for the dsDNA G4 unfolding assay without any further purification. The efficiency of G4 formation was tested by digestion with EcoRI-HF or T7 Endonuclease I (New England Biolabs). 100 ng of DNA was digested with either 5 U of EcoRI-HF (New England Biolabs), or 2 U of T7 Endonuclease I, diluted in 2.1 buffer (New England Biolabs) in a 10 μL reaction incubated at 37°C for 60 min. The products were separated by electrophoresis on a 1% native agarose gel. pUC19-RIR was used as a control. The inverted repeats does not form secondary structure in linearized pUC19-RIR.^73^

### dsDNA G4 unfolding assay

The assay was carried out in a reaction buffer containing 10 mM Tris-HCl pH 7.5, 2 mM ATP, 2 mM dithiothreitol (DTT), 0.25 mg/mL BSA, 2 mM MgCl_2_, 50 mM KCl and 100 ng of linearized G4-DNA in a 15 μL reaction. ATP was omitted in certain reactions as indicated. WT or HIRAN mutant of HLTF proteins were added to the reaction mix on ice and then incubated at 37°C for 30 min. Reactions were subsequently kept at room temperature. Unless indicated otherwise, 4 U of EcoRI-HF diluted in CutSmart buffer (New England Biolabs) was added followed by incubation at 37°C for 30 min. The reactions were terminated by the addition of 1 μL Proteinase K (14-22 mg/ml, Roche) and 5 μL 0.2% stop solution (150 mM EDTA, 0.2% SDS, 30% glycerol, bromophenol blue), and incubated at 37°C for 10 min. The products were separated by electrophoresis in 1% native agarose gels and imaged. The intensities of the bands were quantified using ImageJ. EcoRI-cleaved DNA present in the no protein control reaction was considered as background and subtracted from all the other samples. Reactions with T7 Endonuclease I were carried out similarly.

### Drug dose response assay

500 RPE1 cells were seeded per well in a 96 well plate. After growth for 24 h, PDS was added at the indicated concentration. To monitor proliferation, cells were grown in an IncuCyte Zoom live cell imager (Essen/Sartorius) with images recorded every 4 h for 6 d after addition of the drug. PDS containing media was refreshed 3 d after initial drug addition. The confluence percentage for each image was calculated using the IncuCyte Zoom software over the course of the experiment for each well. For viability measurement, the confluence level at each time point was first normalized to the initial confluence level of the cells before the addition of PDS to account for different initial cell plating density. About 5 d post drug addition, the relative cell proliferation is calculated by dividing the normalized growth of drug treated cells to that of untreated cells. The resulting relative percentage of proliferation and the corresponding drug concentration were fitted to a 4-parameter dose-response curve to calculate IC50 values.

### Western blot analysis

Cells were collected and resuspended in lysis buffer [50 mM Tris-HCl pH 7.5, 150 mM NaCl, 1 mM EDTA, 1% Triton X-100, supplemented with 1x protease inhibitor cocktail (Sigma 11836170001)]. After vortexing at 4°C for 15 min, the lysate was further sonicated using a probe sonicator. Supernatant was collected after high-speed centrifugation. Protein concentration was determined using BCA assay (Thermo Scientific 23227). Equal amounts of total proteins were boiled in Laemmli sample buffer supplemented with 5% beta-mercaptoethanol for 5 min. Proteins were separated by SDS-PAGE and transferred to a PVDF membrane (Millipore IEVH85R). Primary antibodies were: rabbit anti-HLTF (Abcam ab183042 1:5000), mouse anti-MSH2 (abcam ab52266 1:1000), mouse anti-alpha-tubulin (Sigma T9026 1:5000). Secondary antibodies were goat anti-rabbit HRP (Molecular Probes G21234) and goat anti-mouse HRP (Invitrogen 81-6520). Chemi-luminescence was carried out using the Immobilon HRP substrate (Millipore WBKLS0500), and blots were imaged with an Azure cSeries (300) Imager from Azure Biosystems.

## Quantification and Statistical Analysis

All experiments, if not indicated otherwise in the figure legend, were performed three times and representative experiments were depicted. No statistical methods or criteria were used to estimate sample size or to include/exclude samples.

Statistical analysis was performed using Prism (GraphPad Software). Details of how data is presented, including the definition of center (mean or median) and error bars can be found in the figure legends. For all box plots, whiskers indicate the 10th and 90th percentiles, boxes span the 25th to 75th percentiles. Lines inside boxes represent medians.

Details of statistical test for each experiment, including the type of statistical tests used and the number of repeats, can be found in the figure legends. Statistical test results, presented as levels of significance, are shown in the figures. In all cases: ns, not significant; * p < 0.05; ** p < 0.01; *** p < 0.001; **** p < 0.0001.

## Supplementary Material

Supplemental Table 1

Supplemental Table 2

Supplementary Figures

## Data Availability

Original microscopy, gel and western blot images have been deposited at Mendeley and are publicly available as of the date of this publication at the DOI listed in the Key Resource Table. HLTF ChIP-seq data have been deposited at GEO with accession number GEO: GSE247967 and are publicly available as of the date of publication. This study does not report original code. Any additional information required to reanalyze the data reported in this paper is available from the lead contact upon request.

## Highlights

- HLTF is enriched at G4s and suppresses G4 accumulation in all cell cycle phases.
- HLTF translocase directly resolves G4 structures using its ATPase activity.
- HLTF slows replication forks and suppresses PrimPol in response to G4 stabilization.
- HLTF protects cells from DNA damage and growth defects induced by G4 stabilization.

## References

[1] Mirkin EV, Mirkin SM (2007). Replication fork stalling at natural impediments. Microbiol Mol Biol Rev.

[2] Khristich AN, Mirkin SM (2020). On the wrong DNA track: Molecular mechanisms of repeat-mediated genome instability. J Biol Chem.

[3] Brown RE, Freudenreich CH (2021). Structure-forming repeats and their impact on genome stability. Curr Opin Genet Dev.

[4] Wang G, Vasquez KM (2023). Dynamic alternative DNA structures in biology and disease. Nat Rev Genet.

[5] Varshney D, Spiegel J, Zyner K, Tannahill D, Balasubramanian S (2020). The regulation and functions of DNA and RNA G-quadruplexes. Nat Rev Mol Cell Biol.

[6] Sato K, Knipscheer P (2023). G-quadruplex resolution: From molecular mechanisms to physiological relevance. DNA Repair.

[7] Gellert M, Lipsett MN, Davies DR (1962). Helix formation by guanylic acid. Proc Natl Acad Sci USA.

[8] Sen D, Gilbert W (1988). Formation of parallel four-stranded complexes by guanine-rich motifs in DNA and its implications for meiosis. Nature.

[9] Hänsel-Hertsch R, Beraldi D, Lensing SV, Marsico G, Zyner K, Parry A, Di Antonio M, Pike J, Kimura H, Narita M (2016). G-quadruplex structures mark human regulatory chromatin. Nat Genet.

[10] Spiegel J, Cuesta SM, Adhikari S, Hänsel-Hertsch R, Tannahill D, Balasubramanian S (2021). G-quadruplexes are transcription factor binding hubs in human chromatin. Genome Biol.

[11] Shen J, Varshney D, Simeone A, Zhang X, Adhikari S, Tannahill D, Balasubramanian S (2021). Promoter G-quadruplex folding precedes transcription and is controlled by chromatin. Genome Biol.

[12] Vannier J-B, Pavicic-Kaltenbrunner V, Petalcorin MIR, Ding H, Boulton SJ (2012). RTEL1 dismantles T loops and counteracts telomeric G4-DNA to maintain telomere integrity. Cell.

[13] Moye AL, Porter KC, Cohen SB, Phan T, Zyner KG, Sasaki N, Lovrecz GO, Beck JL, Bryan TM (2015). Telomeric G-quadruplexes are a substrate and site of localization for human telomerase. Nat Commun.

[14] Jansson LI, Hentschel J, Parks JW, Chang TR, Lu C, Baral R, Bagshaw CR, Stone MD (2019). Telomere DNA G-quadruplex folding within actively extending human telomerase. Proc Natl Acad Sci USA.

[15] Yang SY, Chang EYC, Lim J, Kwan HH, Monchaud D, Yip S, Stirling PC, Wong JMY (2021). G-quadruplexes mark alternative lengthening of telomeres. NAR Cancer.

[16] Prorok P, Artufel M, Aze A, Coulombe P, Peiffer I, Lacroix L, Guédin A, Mergny J-L, Damaschke J, Schepers A (2019). Involvement of G-quadruplex regions in mammalian replication origin activity. Nat Commun.

[17] Mao S-Q, Ghanbarian AT, Spiegel J, Martínez Cuesta S, Beraldi D, Di Antonio M, Marsico G, Hänsel-Hertsch R, Tannahill D, Balasubramanian S (2018). DNA G-quadruplex structures mold the DNA methylome. Nat Struct Mol Biol.

[18] Sarkies P, Reams C, Simpson LJ, Sale JE (2010). Epigenetic instability due to defective replication of structured DNA. Mol Cell.

[19] Lopes J, Piazza A, Bermejo R, Kriegsman B, Colosio A, Teulade-Fichou M-P, Foiani M, Nicolas A (2011). G-quadruplex-induced instability during leading-strand replication. EMBO J.

[20] Dahan D, Tsirkas I, Dovrat D, Sparks MA, Singh SP, Galletto R, Aharoni A (2018). Pif1 is essential for efficient replisome progression through lagging strand G-quadruplex DNA secondary structures. Nucleic Acids Res.

[21] Lerner LK, Sale JE (2019). Replication of G Quadruplex DNA. Genes.

[22] Belotserkovskii BP, Liu R, Tornaletti S, Krasilnikova MM, Mirkin SM, Hanawalt PC (2010). Mechanisms and implications of transcription blockage by guanine-rich DNA sequences. Proc Natl Acad Sci USA.

[23] Broxson C, Beckett J, Tornaletti S (2011). Transcription arrest by a G quadruplex forming-trinucleotide repeat sequence from the human c-myb gene. Biochemistry.

[24] Belotserkovskii BP, Neil AJ, Saleh SS, Shin JHS, Mirkin SM, Hanawalt PC (2013). Transcription blockage by homopurine DNA sequences: role of sequence composition and single-strand breaks. Nucleic Acids Res.

[25] Rodriguez R, Miller KM, Forment JV, Bradshaw CR, Nikan M, Britton S, Oelschlaegel T, Xhemalce B, Balasubramanian S, Jackson SP (2012). Small-molecule-induced DNA damage identifies alternative DNA structures in human genes. Nat Chem Biol.

[26] De Magis A, Manzo SG, Russo M, Marinello J, Morigi R, Sordet O, Capranico G (2019). DNA damage and genome instability by G-quadruplex ligands are mediated by R loops in human cancer cells. Proc Natl Acad Sci USA.

[27] Puget N, Miller KM, Legube G (2019). Non-canonical DNA/RNA structures during Transcription-Coupled Double-Strand Break Repair: Roadblocks or Bona fide repair intermediates?. DNA Repair.

[28] Georgakopoulos-Soares I, Morganella S, Jain N, Hemberg M, Nik-Zainal S (2018). Noncanonical secondary structures arising from non-B DNA motifs are determinants of mutagenesis. Genome Res.

[29] Liu Y, Zhu X, Wang K, Zhang B, Qiu S (2021). The Cellular Functions and Molecular Mechanisms of G-Quadruplex Unwinding Helicases in Humans. Front Mol Biosci.

[30] Kunkel TA, Erie DA (2005). DNA mismatch repair. Annu Rev Biochem.

[31] Larson ED, Duquette ML, Cummings WJ, Streiff RJ, Maizels N (2005). MutSalpha binds to and promotes synapsis of transcriptionally activated immunoglobulin switch regions. Curr Biol.

[32] Wu Y, Shin-ya K, Brosh RM (2008). FANCJ helicase defective in Fanconia anemia and breast cancer unwinds G-quadruplex DNA to defend genomic stability. Mol Cell Biol.

[33] Sakellariou D, Bak ST, Isik E, Barroso SI, Porro A, Aguilera A, Bartek J, Janscak P, Peña-Diaz J (2022). MutSβ regulates G4-associated telomeric R-loops to maintain telomere integrity in ALT cancer cells. Cell Rep.

[34] Isik E, Shukla K, Pospisilova M, König C, Andrs M, Rao S, Rosano V, Dobrovolna J, Krejci L, Janscak P (2024). MutSβ-MutLβ-FANCJ axis mediates the restart of DNA replication after fork stalling at cotranscriptional G4/R-loops. Sci Adv.

[35] Williams SL, Casas-Delucchi CS, Raguseo F, Guneri D, Li Y, Minamino M, Fletcher EE, Yeeles JT, Keyser UF, Waller ZA (2023). Replication-induced DNA secondary structures drive fork uncoupling and breakage. EMBO J.

[36] Zeman MK, Cimprich KA (2014). Causes and consequences of replication stress. Nat Cell Biol.

[37] Schlacher K, Christ N, Siaud N, Egashira A, Wu H, Jasin M (2011). Double-strand break repair-independent role for BRCA2 in blocking stalled replication fork degradation by MRE11. Cell.

[38] Quinet A, Lemaçon D, Vindigni A (2017). Replication Fork Reversal: Players and Guardians. Mol Cell.

[39] Pasero P, Vindigni A (2017). Nucleases Acting at Stalled Forks: How to Reboot the Replication Program with a Few Shortcuts. Annu Rev Genet.

[40] Rickman K, Smogorzewska A (2019). Advances in understanding DNA processing and protection at stalled replication forks. J Cell Biol.

[41] Berti M, Cortez D, Lopes M (2020). The plasticity of DNA replication forks in response to clinically relevant genotoxic stress. Nat Rev Mol Cell Biol.

[42] Sale JE (2013). Translesion DNA synthesis and mutagenesis in eukaryotes. Cold Spring Harb Perspect Biol.

[43] Guilliam TA, Jozwiakowski SK, Ehlinger A, Barnes RP, Rudd SG, Bailey LJ, Skehel JM, Eckert KA, Chazin WJ, Doherty AJ (2015). Human PrimPol is a highly error-prone polymerase regulated by single-stranded DNA binding proteins. Nucleic Acids Res.

[44] Ding H, Descheemaeker K, Marynen P, Nelles L, Carvalho T, Carmo-Fonseca M, Collen D, Belayew A (1996). Characterization of a helicase-like transcription factor involved in the expression of the human plasminogen activator inhibitor-1 gene. DNA Cell.

[45] Ding H, Benotmane AM, Suske G, Collen D, Belayew A (1999). Functional interactions between Sp1 or Sp3 and the helicase-like transcription factor mediate basal expression from the human plasminogen activator inhibitor-1 gene. J Biol Chem.

[46] Dungrawala H, Rose KL, Bhat KP, Mohni KN, Glick GG, Couch FB, Cortez D (2015). The Replication Checkpoint Prevents Two Types of Fork Collapse without Regulating Replisome Stability. Mol Cell.

[47] Kile AC, Chavez DA, Bacal J, Eldirany S, Korzhnev DM, Bezsonova I, Eichman BF, Cimprich KA (2015). HLTF’s ancient HIRAN domain binds 3’ DNA ends to drive replication fork reversal. Mol Cell.

[48] Wessel SR, Mohni KN, Luzwick JW, Dungrawala H, Cortez D (2019). Functional Analysis of the Replication Fork Proteome Identifies BET Proteins as PCNA Regulators. Cell Rep.

[49] Unk I, Hajdú I, Fátyol K, Hurwitz J, Yoon J-H, Prakash L, Prakash S, Haracska L (2008). Human HLTF functions as a ubiquitin ligase for proliferating cell nuclear antigen polyubiquitination. Proc Natl Acad Sci USA.

[50] Krijger PHL, Lee K-Y, Wit N, van den Berk PCM, Wu X, Roest HP, Maas A, Ding H, Hoeijmakers JHJ, Myung K (2011). HLTF and SHPRH are not essential for PCNA polyubiquitination, survival and somatic hypermutation: existence of an alternative E3 ligase. DNA Repair.

[51] Lin J-R, Zeman MK, Chen J-Y, Yee M-C, Cimprich KA (2011). SHPRH and HLTF act in a damage-specific manner to coordinate different forms of postreplication repair and prevent mutagenesis. Mol Cell.

[52] Masuda Y, Suzuki M, Kawai H, Hishiki A, Hashimoto H, Masutani C, Hishida T, Suzuki F, Kamiya K (2012). En bloc transfer of polyubiquitin chains to PCNA in vitro is mediated by two different human E2-E3 pairs. Nucleic Acids Res.

[53] Blastyák A, Hajdú I, Unk I, Haracska L (2010). Role of double-stranded DNA translocase activity of human HLTF in replication of damaged DNA. Mol Cell Biol.

[54] Achar YJ, Balogh D, Haracska L (2011). Coordinated protein and DNA remodeling by human HLTF on stalled replication fork. Proc Natl Acad Sci USA.

[55] Hishiki A, Hara K, Ikegaya Y, Yokoyama H, Shimizu T, Sato M, Hashimoto H (2015). Structure of a Novel DNA-binding Domain of Helicase-like Transcription Factor (HLTF) and Its Functional Implication in DNA Damage Tolerance. J Biol Chem.

[56] Chavez DA, Greer BH, Eichman BF (2018). The HIRAN domain of helicase-like transcription factor positions the DNA translocase motor to drive efficient DNA fork regression. J Biol Chem.

[57] Bai G, Kermi C, Stoy H, Schiltz CJ, Bacal J, Zaino AM, Hadden MK, Eichman BF, Lopes M, Cimprich KA (2020). HLTF Promotes Fork Reversal, Limiting Replication Stress Resistance and Preventing Multiple Mechanisms of Unrestrained DNA Synthesis. Mol Cell.

[58] van Toorn M, Turkyilmaz Y, Han S, Zhou D, Kim HS, Salas-Armenteros I, Kim M, Akita M, Wienholz F, Raams A (2022). Active DNA damage eviction by HLTF stimulates nucleotide excision repair. Mol Cell.

[59] Burkovics P, Sebesta M, Balogh D, Haracska L, Krejci L (2014). Strand invasion by HLTF as a mechanism for template switch in fork rescue. Nucleic Acids Res.

[60] Dhont L, Mascaux C, Belayew A (2016). The helicase-like transcription factor (HLTF) in cancer: loss of function or oncomorphic conversion of a tumor suppressor? Cell. Mol Life Sci.

[61] Sandhu S, Wu X, Nabi Z, Rastegar M, Kung S, Mai S, Ding H (2012). Loss of HLTF function promotes intestinal carcinogenesis. Mol Cancer.

[62] Moinova HR, Chen W-D, Shen L, Smiraglia D, Olechnowicz J, Ravi L, Kasturi L, Myeroff L, Plass C, Parsons R (2002). HLTF gene silencing in human colon cancer. Proc Natl Acad Sci USA.

[63] Hamai Y, Oue N, Mitani Y, Nakayama H, Ito R, Matsusaki K, Yoshida K, Toge T, Yasui W (2003). DNA hypermethylation and histone hypoacetylation of the HLTF gene are associated with reduced expression in gastric carcinoma. Cancer Sci.

[64] Liu L, Liu H, Zhou Y, He J, Liu Q, Wang J, Zeng M, Yuan D, Tan F, Zhou Y (2018). HLTF suppresses the migration and invasion of colorectal cancer cells via TGF-β/SMAD signaling in vitro. Int J Oncol.

[65] Sirbu BM, McDonald WH, Dungrawala H, Badu-Nkansah A, Kavanaugh GM, Chen Y, Tabb DL, Cortez D (2013). Identification of proteins at active, stalled, and collapsed replication forks using isolation of proteins on nascent DNA (iPOND) coupled with mass spectrometry. J Biol Chem.

[66] Duquette ML, Handa P, Vincent JA, Taylor AF, Maizels N (2004). Intracellular transcription of G-rich DNAs induces formation of G-loops, novel structures containing G4 DNA. Genes Dev.

[67] Crossley MP, Bocek M, Cimprich KA (2019). R-Loops as Cellular Regulators and Genomic Threats. Mol Cell.

[68] Petermann E, Lan L, Zou L (2022). Sources, resolution and physiological relevance of R-loops and RNA-DNA hybrids. Nat Rev Mol Cell Biol.

[69] Owen BAL, Yang Z, Lai M, Gajec M, Badger JD, Hayes JJ, Edelmann W, Kucherlapati R, Wilson TM, McMurray CT (2005). (CAG)(n)-hairpin DNA binds to Msh2-Msh3 and changes properties of mismatch recognition. Nat Struct Mol Biol.

[70] Young SJ, Sebald M, Shah Punatar R, Larin M, Masino L, Rodrigo-Brenni MC, Liang C-C, West SC (2020). MutSβ Stimulates Holliday Junction Resolution by the SMX Complex. Cell Rep.

[71] Burdova K, Mihaljevic B, Sturzenegger A, Chappidi N, Janscak P (2015). The Mismatch-Binding Factor MutSβ Can Mediate ATR Activation in Response to DNA Double-Strand Breaks. Mol Cell.

[72] McKinney JA, Wang G, Mukherjee A, Christensen L, Subramanian SHS, Zhao J, Vasquez KM (2020). Distinct DNA repair pathways cause genomic instability at alternative DNA structures. Nat Commun.

[73] Mengoli V, Ceppi I, Sanchez A, Cannavo E, Halder S, Scaglione S, Gaillard P-H, McHugh PJ, Riesen N, Pettazzoni P (2023). WRN helicase and mismatch repair complexes independently and synergistically disrupt cruciform DNA structures. EMBO J.

[74] Orlando DA, Chen MW, Brown VE, Solanki S, Choi YJ, Olson ER, Fritz CC, Bradner JE, Guenther MG (2014). Quantitative ChIP-Seq normalization reveals global modulation of the epigenome. Cell Rep.

[75] Hui WWI, Simeone A, Zyner KG, Tannahill D, Balasubramanian S (2021). Single-cell mapping of DNA G-quadruplex structures in human cancer cells. Sci Rep.

[76] Marsico G, Chambers VS, Sahakyan AB, McCauley P, Boutell JM, Antonio MD, Balasubramanian S (2019). Whole genome experimental maps of DNA G-quadruplexes in multiple species. Nucleic Acids Res.

[77] Acurzio B, Cecere F, Giaccari C, Verma A, Russo R, Valletta M, Hay Mele B, Angelini C, Chambery A, Riccio A (2022). The mismatch-repair proteins MSH2 and MSH6 interact with the imprinting control regions through the ZFP57-KAP1 complex. Epigenetics Chromatin.

[78] Lyu J, Shao R, Kwong Yung PY, Elsässer SJ (2022). Genome-wide mapping of G-quadruplex structures with CUT&Tag. Nucleic Acids Res.

[79] Tan J, Wang X, Phoon L, Yang H, Lan L (2020). Resolution of ROS-induced G-quadruplexes and R-loops at transcriptionally active sites is dependent on BLM helicase. FEBS Lett.

[80] Lim G, Hohng S (2020). Single-molecule fluorescence studies on cotranscriptional G-quadruplex formation coupled with R-loop formation. Nucleic Acids Res.

[81] Crossley MP, Brickner JR, Song C, Zar SMT, Maw SS, Chédin F, Tsai M-S, Cimprich KA (2021). Catalytically inactive, purified RNase H1: A specific and sensitive probe for RNA-DNA hybrid imaging. J Cell Biol.

[82] Garcia-Barcena C, Osinalde N, Ramirez J, Mayor U (2020). How to Inactivate Human Ubiquitin E3 Ligases by Mutation. Front Cell Dev Biol.

[83] Yusufzai T, Kadonaga JT (2008). HARP is an ATP-driven annealing helicase. Science.

[84] Arora R, Lee Y, Wischnewski H, Brun CM, Schwarz T, Azzalin CM (2014). RNaseH1 regulates TERRA-telomeric DNA hybrids and telomere maintenance in ALT tumour cells. Nat Commun.

[85] Santos-Pereira JM, Aguilera A (2015). R loops: new modulators of genome dynamics and function. Nat Rev Genet.

[86] García-Muse T, Aguilera A (2019). R Loops: From Physiological to Pathological Roles. Cell.

[87] Amato R, Valenzuela M, Berardinelli F, Salvati E, Maresca C, Leone S, Antoccia A, Sgura A (2020). G-quadruplex Stabilization Fuels the ALT Pathway in ALT-positive Osteosarcoma Cells. Genes.

[88] Barroso-González J, García-Expósito L, Galaviz P, Lynskey ML, Allen JAM, Hoang S, Watkins SC, Pickett HA, O’Sullivan RJ (2021). Anti-recombination function of MutSα restricts telomere extension by ALT-associated homology-directed repair. Cell Rep.

[89] Lezaja A, Panagopoulos A, Wen Y, Carvalho E, Imhof R, Altmeyer M (2021). RPA shields inherited DNA lesions for post-mitotic DNA synthesis. Nat Commun.

[90] Zhang J-M, Zou L (2020). Alternative lengthening of telomeres: from molecular mechanisms to therapeutic outlooks. Cell Biosci.

[91] Loe TK, Li JSZ, Zhang Y, Azeroglu B, Boddy MN, Denchi EL (2020). Telomere length heterogeneity in ALT cells is maintained by PML-dependent localization of the BTR complex to telomeres. Genes Dev.

[92] Kumar C, Batra S, Griffith JD, Remus D (2021). The interplay of RNA:DNA hybrid structure and G-quadruplexes determines the outcome of R-loop-replisome collisions. Elife.

[93] Schiavone D, Jozwiakowski SK, Romanello M, Guilbaud G, Guilliam TA, Bailey LJ, Sale JE, Doherty AJ (2016). PrimPol Is Required for Replicative Tolerance of G Quadruplexes in Vertebrate Cells. Mol Cell.

[94] Gan W, Guan Z, Liu J, Gui T, Shen K, Manley JL, Li X (2011). R-loop-mediated genomic instability is caused by impairment of replication fork progression. Genes Dev.

[95] Xu H, Di Antonio M, McKinney S, Mathew V, Ho B, O’Neil NJ, Santos ND, Silvester J, Wei V, Garcia J (2017). CX-5461 is a DNA G-quadruplex stabilizer with selective lethality in BRCA1/2 deficient tumours. Nat Commun.

[96] Hodson C, van Twest S, Dylewska M, O’Rourke JJ, Tan W, Murphy VJ, Walia M, Abbouche L, Nieminuszczy J, Dunn E (2022). Branchpoint translocation by fork remodelers as a general mechanism of R-loop removal. Cell Rep.

[97] Déjardin J, Kingston RE (2009). Purification of proteins associated with specific genomic Loci. Cell.

[98] Leuzzi G, Vasciaveo A, Taglialatela A, Chen X, Firestone TM, Hickman AR, Mao W, Thakar T, Vaitsiankova A, Huang J-W (2024). SMARCAL1 is a dual regulator of innate immune signaling and PD-L1 expression that promotes tumor immune evasion. Cell.

[99] Frizzell A, Nguyen JHG, Petalcorin MIR, Turner KD, Boulton SJ, Freudenreich CH, Lahue RS (2016). RTEL1 Inhibits Trinucleotide Repeat Expansions and Fragility. Cell Rep.

[100] Rastokina A, Cebrián J, Mozafari N, Mandel NH, Smith CIE, Lopes M, Zain R, Mirkin SM (2023). Large-scale expansions of Friedreich’s ataxia GAA•TTC repeats in an experimental human system: role of DNA replication and prevention by LNA-DNA oligonucleotides and PNA oligomers. Nucleic Acids Res.

[101] Saydam N, Kanagaraj R, Dietschy T, Garcia PL, Peña-Diaz J, Shevelev I, Stagljar I, Janscak P (2007). Physical and functional interactions between Werner syndrome helicase and mismatch-repair initiation factors. Nucleic Acids Res.

[102] Yang Q, Zhang R, Wang XW, Linke SP, Sengupta S, Hickson ID, Pedrazzi G, Perrera C, Stagljar I, Littman SJ (2004). The mismatch DNA repair heterodimer, hMSH2/6, regulates BLM helicase. Oncogene.

[103] Peng M, Xie J, Ucher A, Stavnezer J, Cantor SB (2014). Crosstalk between BRCA-Fanconi anemia and mismatch repair pathways prevents MSH2-dependent aberrant DNA damage responses. EMBO J.

[104] Peng M, Cong K, Panzarino NJ, Nayak S, Calvo J, Deng B, Zhu LJ, Morocz M, Hegedus L, Haracska L (2018). Opposing Roles of FANCJ and HLTF Protect Forks and Restrain Replication during Stress. Cell Rep.

[105] Tyanova S, Temu T, Sinitcyn P, Carlson A, Hein MY, Geiger T, Mann M, Cox J (2016). The Perseus computational platform for comprehensive analysis of (prote)omics data. Nat Methods.

[106] Schindelin J, Arganda-Carreras I, Frise E, Kaynig V, Longair M, Pietzsch T, Preibisch S, Rueden C, Saalfeld S, Schmid B (2012). Fiji: an open-source platform for biological-image analysis. Nat Methods.

[107] Spencley AL, Bar S, Swigut T, Flynn RA, Lee C-H, Chen LF, Bassik MC, Wysocka J (2023). Co-transcriptional genome surveillance by HUSH is coupled to termination machinery. Mol Cell.

[108] Langmead B, Salzberg SL (2012). Fast gapped-read alignment with Bowtie 2. Nat Methods.

[109] Li H, Handsaker B, Wysoker A, Fennell T, Ruan J, Homer N, Marth G, Abecasis G, Durbin R, 1000 Genome Project Data Processing Subgroup (2009). The Sequence Alignment/Map format and SAMtools. Bioinformatics.

[110] Amemiya HM, Kundaje A, Boyle AP (2019). The ENCODE Blacklist: Identification of Problematic Regions of the Genome. Sci Rep.

[111] Ramírez F, Ryan DP, Grüning B, Bhardwaj V, Kilpert F, Richter AS, Heyne S, Dündar F, Manke T (2016). deepTools2: a next generation web server for deep-sequencing data analysis. Nucleic Acids Res.

[112] Zhang Y, Liu T, Meyer CA, Eeckhoute J, Johnson DS, Bernstein BE, Nusbaum C, Myers RM, Brown M, Li W (2008). Model-based analysis of ChIP-Seq (MACS. Genome Biol.

[113] Landt SG, Marinov GK, Kundaje A, Kheradpour P, Pauli F, Batzoglou S, Bernstein BE, Bickel P, Brown JB, Cayting P (2012). ChIP-seq guidelines and practices of the ENCODE and modENCODE consortia. Genome Res.

[114] Quinlan AR (2014). BEDTools: The Swiss-Army Tool for Genome Feature Analysis. Curr Protoc Bioinformatics.

[115] Heinz S, Benner C, Spann N, Bertolino E, Lin YC, Laslo P, Cheng JX, Murre C, Singh H, Glass CK (2010). Simple combinations of lineage-determining transcription factors prime cis-regulatory elements required for macrophage and B cell identities. Mol Cell.

[116] Campbell SJ, Edwards RA, Leung CCY, Neculai D, Hodge CD, Dhe-Paganon S, Glover JNM (2012). Molecular insights into the function of RING finger (RNF)-containing proteins hRNF8 and hRNF168 in Ubc13/Mms2-dependent ubiquitylation. J Biol Chem.

[117] Halder S, Ranjha L, Taglialatela A, Ciccia A, Cejka P (2022). Strand annealing and motor driven activities of SMARCAL1 and ZRANB3 are stimulated by RAD51 and the paralog complex. Nucleic Acids Res.

[118] Kiianitsa K, Solinger JA, Heyer W-D (2003). NADH-coupled microplate photometric assay for kinetic studies of ATP-hydrolyzing enzymes with low and high specific activities. Anal Biochem.

[119] Wallgren M, Mohammad JB, Yan K-P, Pourbozorgi-Langroudi P, Ebrahimi M, Sabouri N (2016). G-rich telomeric and ribosomal DNA sequences from the fission yeast genome form stable G-quadruplex DNA structures in vitro and are unwound by the Pfh1 DNA helicase. Nucleic Acids Res.

[120] Vernekar DV, Reginato G, Adam C, Ranjha L, Dingli F, Marsolier M-C, Loew D, Guérois R, Llorente B, Cejka P (2021). The Pif1 helicase is actively inhibited during meiotic recombination which restrains gene conversion tract length. Nucleic Acids Res.

[121] Amrane S, Adrian M, Heddi B, Serero A, Nicolas A, Mergny J-L, Phan AT (2012). Formation of pearl-necklace monomorphic G-quadruplexes in the human CEB25 minisatellite. J Am Chem Soc.

