# Supplementary material for: HLTF Resolves G4s and Promotes G4-Induced Replication Fork Slowing to Maintain Genome Stability
Source: Mol Cell. Author manuscript; Available in PMC 2024 Sep 18. (PMC11366124; doi:10.1016/j.molcel.2024.07.018)
Supplement: Supplementary Figures [file EMS198630-supplement-Supplementary_Figures.pdf]

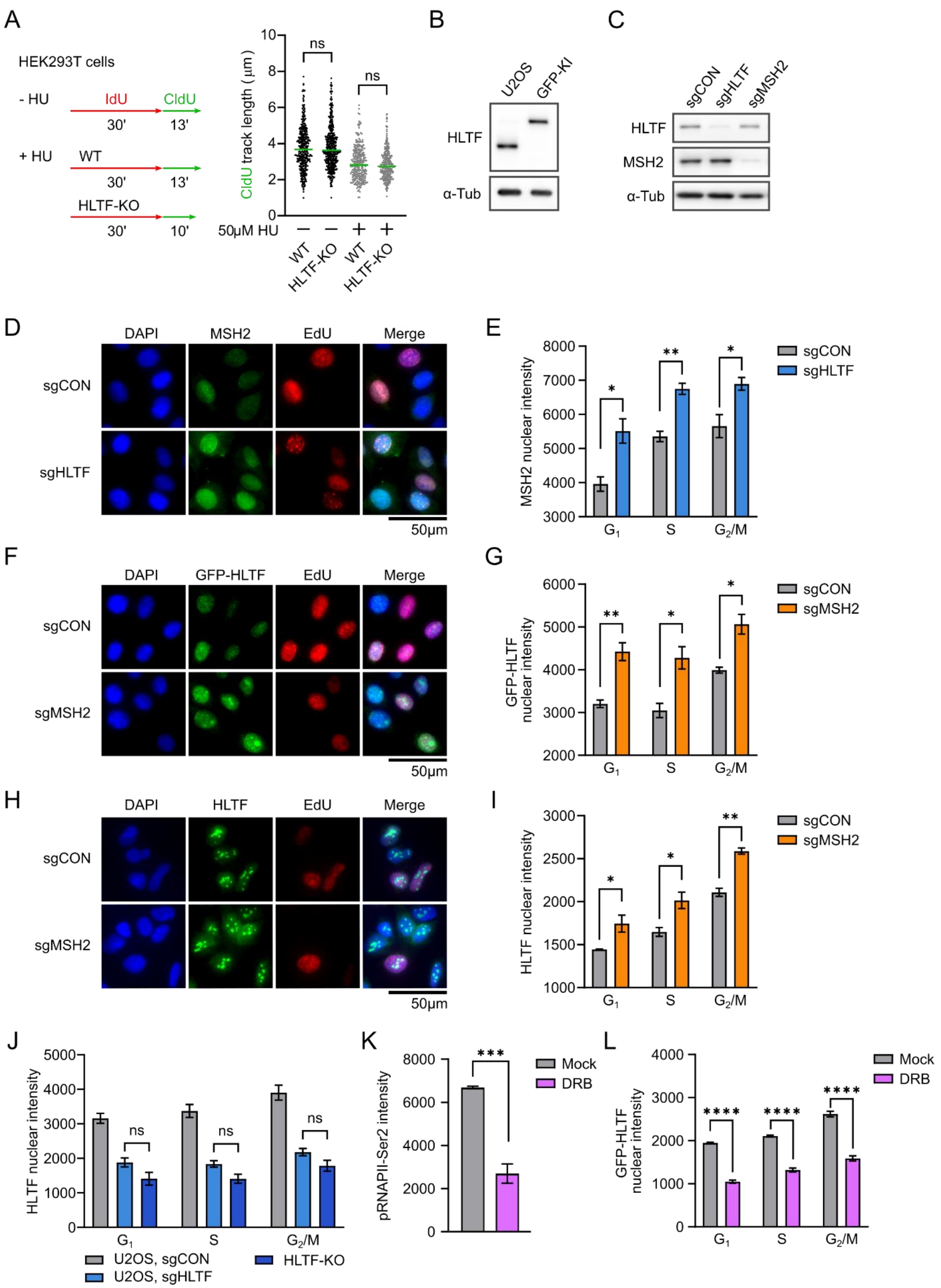

Figure S1. The cell cycle independent regulation of HLTF chromatin binding by MSH2 and transcription. Related to Figure 1.

A. Left: experiment setup of replication fork progression assay. Right: dot plot of CldU tract lengths (n = 3). Line represents median. Mann-Whitney test was performed.
B. Western blot of WT and GFP-HLTF U2OS cells. An HLTF antibody was used.
C. Western blot in GFP-HLTF U2OS cells 96h after sgRNA transfection.
D. Representative IF images in U2OS cells expressing GFP-HLTF 96h after sgRNA transfection.
E. Mean chromatin-bound MSH2 intensity in U2OS cells expressing GFP-HLTF, as shown in D. The median of the mean MSH2 intensity is used to calculate the mean ± SEM (n=3).
F. Representative IF images in U2OS cells expressing GFP-HLTF 96h after sgRNA transfection. A GFP antibody was used to detect GFP-HLTF.
G. Mean chromatin-bound GFP-HLTF intensity in U2OS cells expressing GFP-HLTF, as shown in F. The median of the mean GFP intensity is used to calculate the mean ± SEM (n=3).
H. Representative IF images in U2OS cells 96h after sgRNA transfection. An HLTF antibody was used to detect HLTF.
I. Mean chromatin-bound HLTF intensity in U2OS cells, as shown in H. The median of the mean HLTF intensity is used to calculate the mean ± SEM (n=3).
J. Mean chromatin-bound HLTF intensity in WT and HLTF-KO U2OS cells 96h after sgRNA transfection. The median of the mean HLTF intensity is used to calculate the mean ± SEM (n=3). An HLTF antibody was used to detect HLTF.
K. Mean phosphorylated Ser2 of RNA polymerase II C-terminal domain levels (pRNAPII-Ser2) in U2OS cells expressing GFP-HLTF after 4h of mock or DRB (100 µM) treatment. The median of the mean pRNAPII-Ser2 intensity is used to calculate the mean ± SEM (n=4).
L. Mean chromatin-bound HLTF intensity in U2OS cells expressing GFP-HLTF after 4h of mock or DRB (100 µM) treatment. A GFP antibody was used for IF detection of GFP-HLTF. The median of the mean GFP-HLTF intensity is used to calculate the mean ± SEM (n=4). T-tests were performed in this figure.

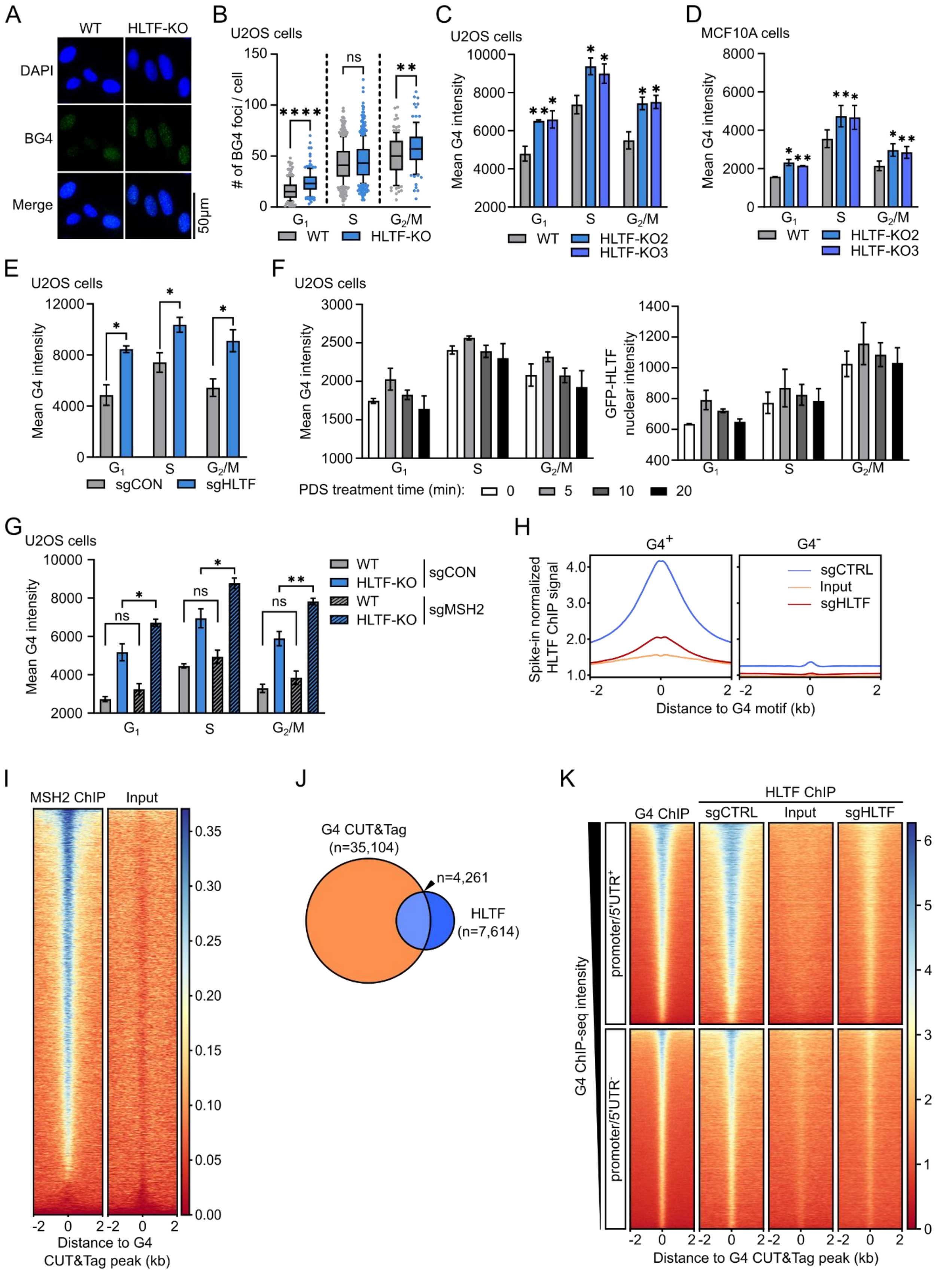

Figure S2. HLTF suppresses G4 accumulation in cells and is enriched at G4 structures in the human genome. Related to Figure 2.

A. Representative IF images in WT and HLTF-KO U2OS cells. G4 is detected using the BG4 antibody.
B. Average number of G4 foci/cell in WT and HLTF-KO U2OS cells, as shown in A. Mann-Whitney test was performed.
C. Mean G4 intensity in WT and HLTF-KO U2OS cells. The median of the mean G4 intensity is used to calculate the mean ± SEM (n=3).
D. Mean G4 intensity in WT and HLTF-KO MCF10A cells. The median of the mean G4 intensity is used to calculate the mean ± SEM (n=3). In C and D, one-way ANOVA was performed followed by Dunnett's test. Statistical test results compared to the WT cells are shown.
E. Mean G4 intensity in U2OS cells after sgRNA transfection. The median of the mean G4 intensity is used to calculate the mean ± SEM (n=3). T-test was performed.
F. Mean G4 (left) and chromatin-bound HLTF (right) intensity in U2OS cells expressing GFP-HLTF after PDS (10 µM) treatment for the indicated time (min). The median of the mean G4 intensity is used to calculate the mean ± SEM (n=3).
G. Mean G4 intensity in WT and HLTF-KO U2OS cells after sgRNA transfection. The median of the mean G4 intensity is used to calculate the mean ± SEM (n=3). T-test was performed.
H. Aggregate plot showing HLTF ChIP-seq coverage (y-axis) relative to the distance (x-axis) from G4 motifs (GSE110582) identified in the human genome *in vitro* in kb. G4 motifs are segregated based on whether they are within G4 structures identified in U2OS cells by G4 CUT&Tag: G4 motifs found in G4 CUT&Tag peaks are $G4^{+}$ (n=55,768) or otherwise $G4^{-}$ (n=1,207,711).
I. Heatmaps showing ChIP-seq coverage for MSH2 (GSE205043) at G4 CUT&Tag peaks (GSE173103) in mESCs (E14). The x-axis represents the distance from the G4 CUT&Tag peak in kb.
J. Venn diagram showing the number of G4 CUT&Tag, HLTF ChIP-seq, and overlapping peaks.
K. Heatmaps showing ChIP-seq coverage for G4 and HLTF at G4 CUT&Tag peaks. The x-axis represents the distance from the G4 CUT&Tag peak in kb. G4 peaks are segregated based on whether they are within the promoter or 5'UTR. Promoters are identified as ±1kb of the annotated TSS. Heatmaps were sorted by G4 ChIP-seq signal intensity for all ChIP-seq samples. Spearman correlation coefficient between G4 and HLTF ChIP-seq signal (sgCON) was 0.64 for G4 peaks in promoter or 5'UTR ($promoter/5'UTR^{+}$), and 0.70 for G4 peaks out of promoter or 5'UTR ($promoter/5'UTR^{-}$). Related to Figure 2I.

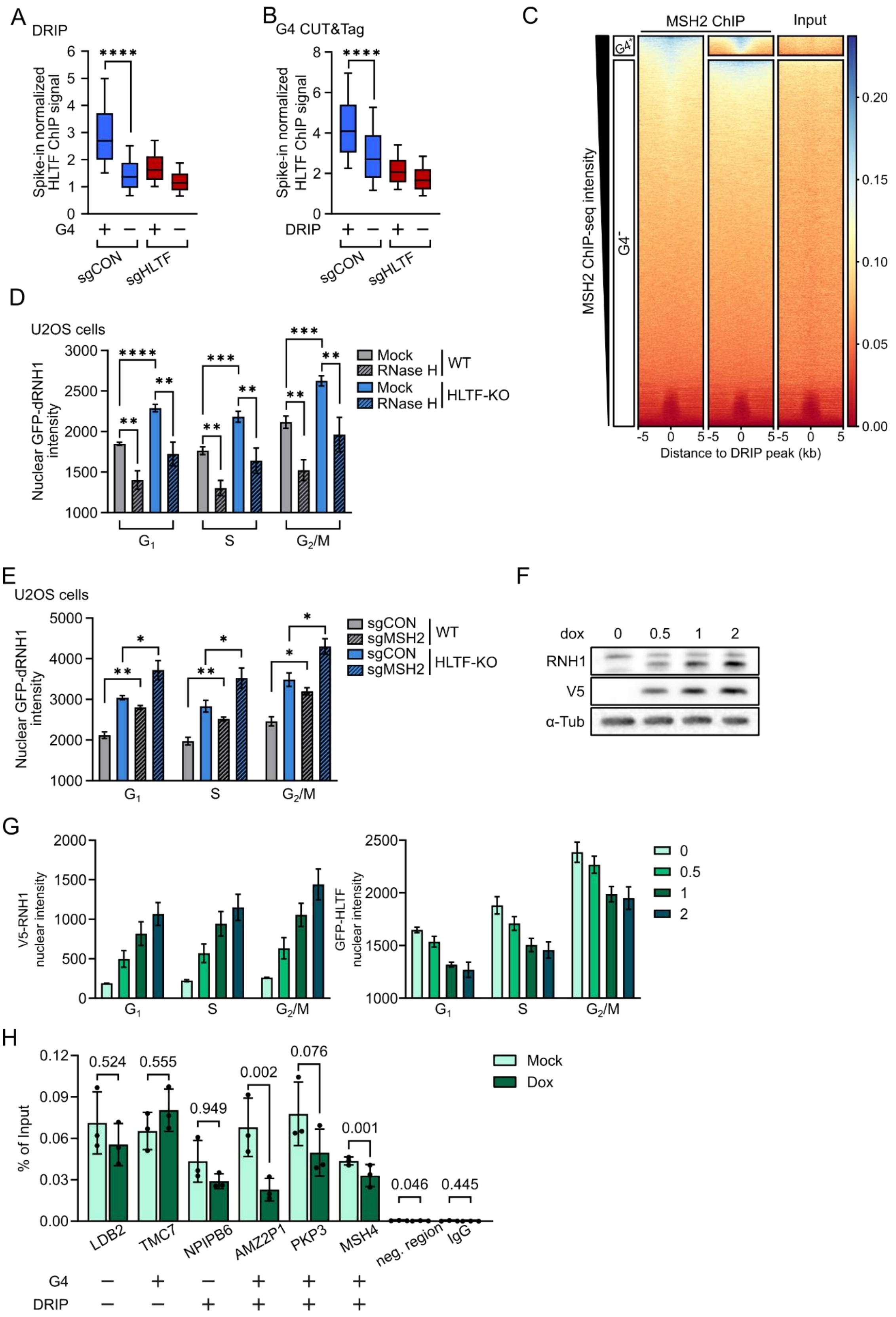

Figure S3. HLTF is enriched at RNA-DNA hybrids stabilized by G4s. Related to Figure 3.

A. Mean HLTF ChIP-seq signal intensity in all RNA-DNA hybrid peaks identified by DRIP-seq in U2OS cells. DRIP peaks are segregated based on whether they overlap with G4 peaks identified by G4 CUT&Tag: DRIP peaks that overlap with G4 peaks are $G4^+$ (n=11,499) or otherwise $G4^-$ (n=59,090).
B. Mean HLTF ChIP-seq signal intensity in all G4 CUT&Tag peaks identified in U2OS cells. G4 peaks are segregated based on if they overlap with RNA-DNA hybrid peaks identified by DRIP-seq: G4 peaks that overlap with DRIP peaks are $DRIP^+$ (n=10,792) or otherwise $DRIP^-$ (n=24,312).
C. Heatmaps showing ChIP-seq coverage for MSH2 at RNA-DNA hybrid peaks identified in mESCs (E14) by DRIP-seq (GSE70189). The x-axis represents the distance from the DRIP peak in kb. DRIP peaks are segregated based on whether they overlap with G4 peaks identified by G4 CUT&Tag: DRIP peaks that overlap with G4 peaks are $G4^+$(n=2,693) or otherwise $G4^-$ (n=51,117).
D. Mean nuclear RNA-DNA hybrid intensity in WT and HLTF-KO U2OS cells after RNase H digestion. The median of the mean nuclear RNA-DNA hybrid intensity is used to calculate the mean ± SEM (n=3).
E. Mean nuclear RNA-DNA hybrid intensity in WT and HLTF-KO U2OS cells after sgRNA transfection. The median of the mean nuclear RNA-DNA hybrid intensity is used to calculate the mean ± SEM (n=3). All statistical tests in this figure are t-tests.
F. Western blot of V5-tagged RNH1 expressed in GFP-HLTF U2OS cells.
G. Mean nuclear V5-RNH1 (left) and GFP-HLTF (right) intensity in U2OS cells expressing GFP-HLTF after 24 h of dox (0, 0.5, 1 and 2 µg/mL) induction. The median of the mean nuclear V5-RNH1 and GFP-HLTF intensity is used to calculate the mean ± SEM (n=3).
H. Representative GFP- HLTF ChIP-qPCR results of U2OS cells expressing GFP-HLTF after mock or dox (1 µg/mL) RNaseH-induction for 24 h. Loci where HLTF peaks were identified were selected to design qPCR primers. HLTF peaks were further classified based on whether they were in the proximity of G4 CUT&Tag or DRIP peaks. n=3 technical replicates of two independent experiments were first normalized to the mock samples. A t-test was performed on the normalized data. p-values are shown. An HLTF unbound locus (neg. region) and IgG control served as controls.

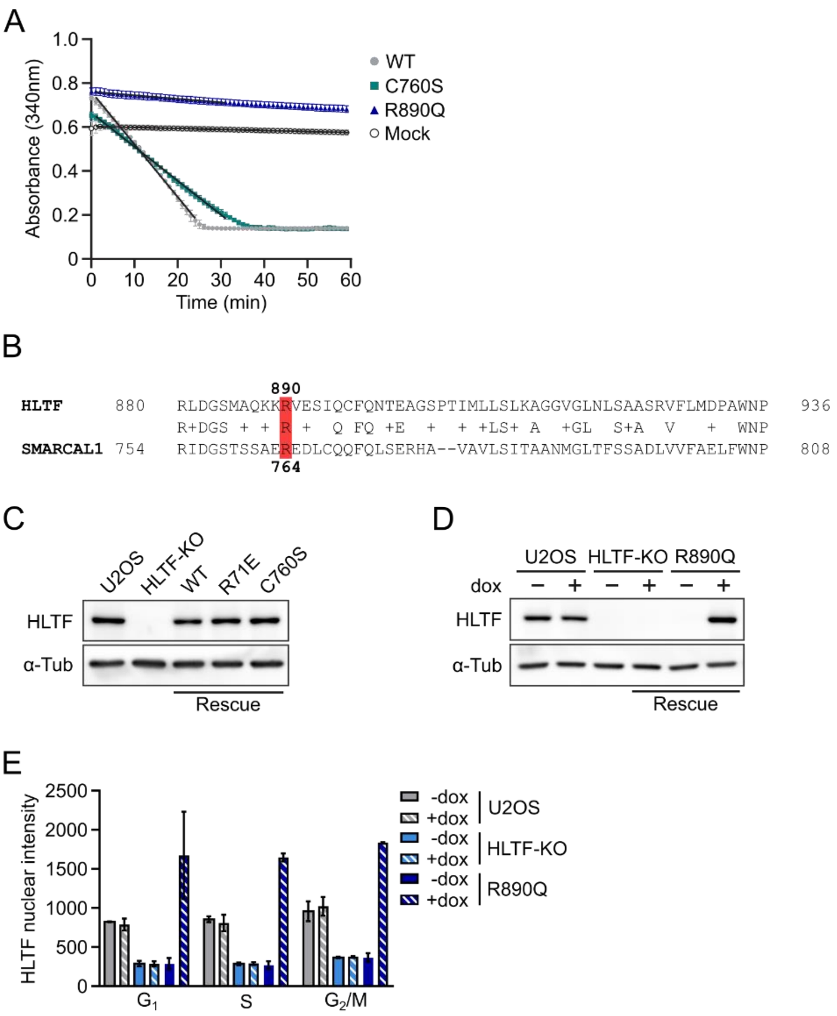

Figure S4. HLTF suppresses G4s in an ATPase-dependent manner in cells. Related to Figure 4.

A. Quantification of NADH-coupled regenerative ATPase activity. Slopes used to calculate the ATPase rates shown in Figure 4C are shown as black lines and were determined by linear least squares regression of the linear range of each trace. Mock, no-enzyme control used to calculate background NADH decomposition. Data are plotted as the mean ± SD (n=3).
B. Protein sequence alignment of HLTF and SMARCAL1 at their C-terminus. HLTF's R890 and SMARCAL1's R764 residues are highlighted in red.
C. Western blot in U2OS WT and HLTF-KO cells expressing HLTF WT or mutant proteins.
D. Western blot in U2OS WT and HLTF-KO cells expressing the HLTF R890Q mutant, after 24 h of dox (500 ng/mL) induction. Note that no R890Q mutant expression was observed in the absence of dox. See method details.
E. Mean chromatin-bound HLTF levels in WT and HLTF-KO U2OS cells and HLTF-KO cells expressing the R890Q mutant after 24 h of dox (500 ng/mL) induction. A HLTF antibody was used for HLTF detection.

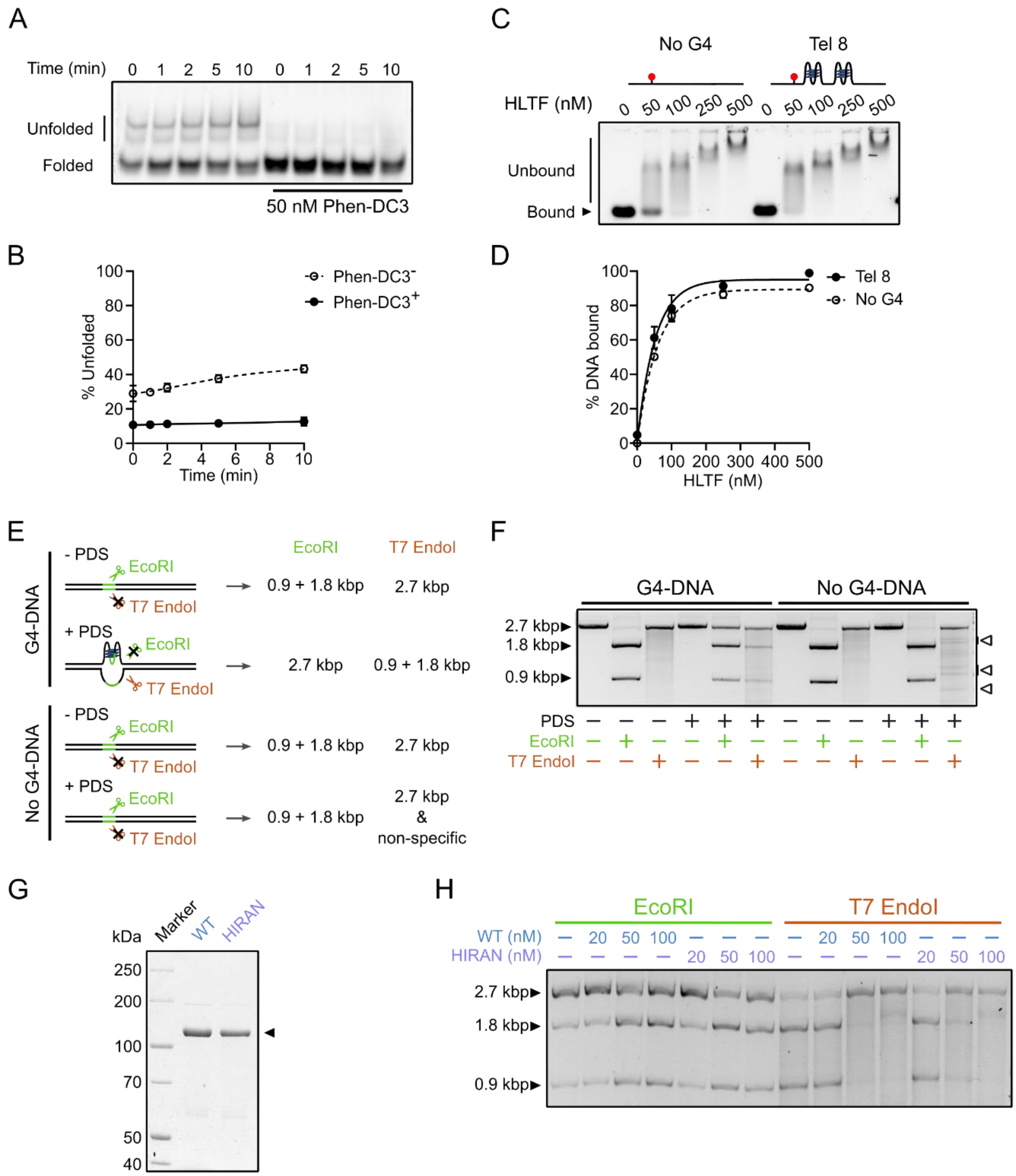

Figure S5. HLTF promotes ATP-dependent G4 unfolding in dsDNA. Related to Figure 5.

A. Native gel images showing spontaneous ssDNA G4 unfolding. Phen-DC3 prevents unfolding.
B. Quantification of spontaneous ssDNA G4 unfolding. Related to A. The Phen-DC3$^-$ sample is the same as the HLTF$^-$ sample shown in Figure 5C. Data are plotted as the mean ± SEM (n=3).

C. Top: schematic of ssDNA substrates used in ssDNA G4 binding assay. Bottom: gel image showing mobility shift of the ssDNA substrates bound by increasing concentrations of HLTF protein.
D. Quantification of HLTF binding to ssDNA that can (Tel 8) or cannot (No G4) form G4s. Data are plotted as the mean ± SEM (n=3).
E. Schematic of the validation of DNA substrates used in the dsDNA G4 unfolding assay. PDS was used at 3 μM.
F. Gel image showing the validation of DNA substrates used in the dsDNA G4 unfolding assay. Related to E.
G. Gel image showing the purified recombinant HLTF WT and a HIRAN (N90A,N91A) mutant used in dsDNA G4 unfolding assays. See also in Figure 5E, F.
H. Representative gel image showing the processing of the G4 dsDNA substrate by HLTF WT and a HIRAN (N90A, N91A) mutant. After the reaction, the substrate was digested with either EcoRI or T7 Endonuclease I.

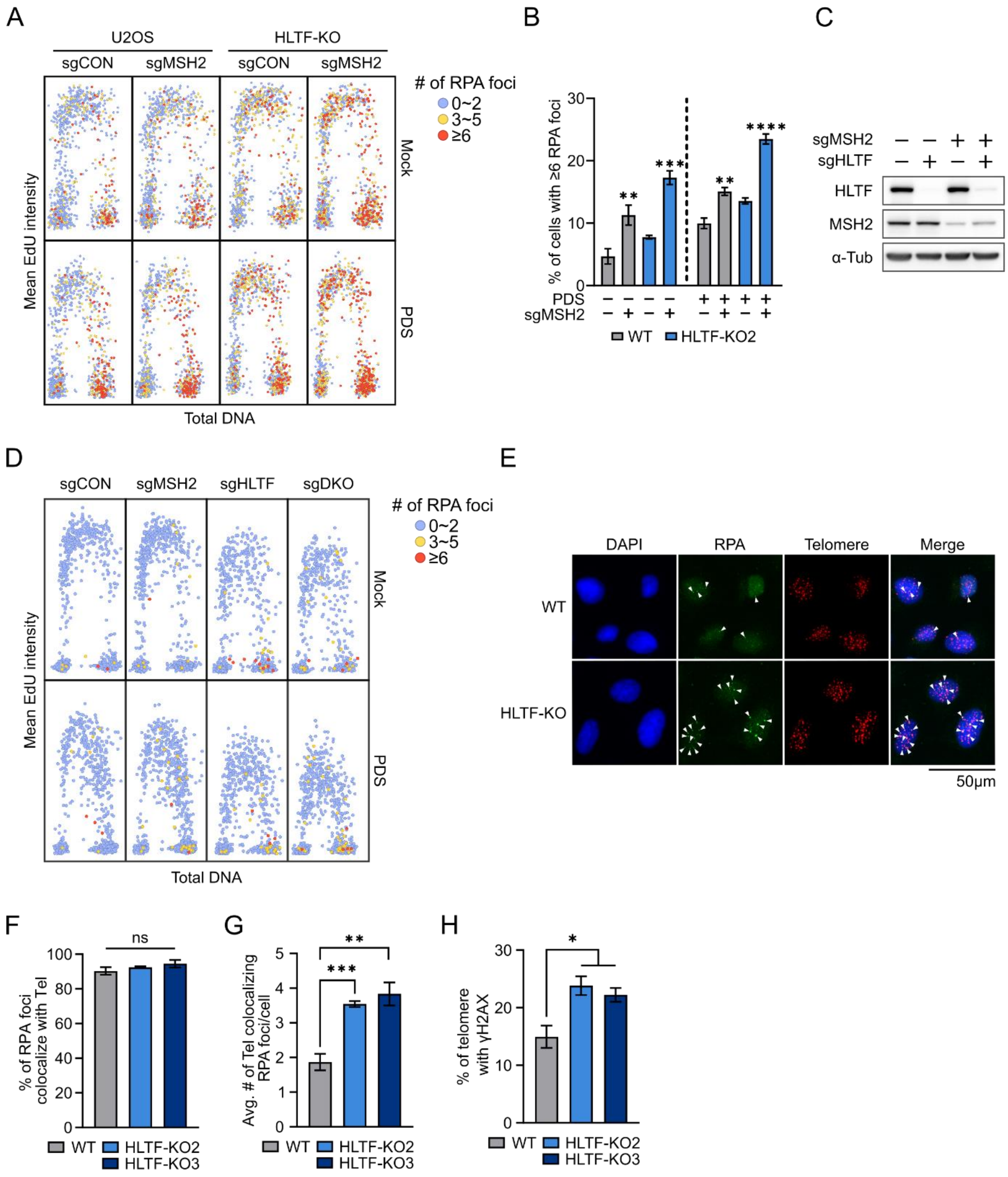

Figure S6. HLTF suppresses ALT activity in an ATPase-dependent manner. Related to Figure 6.

A. Scatter plots of total DNA content versus mean EdU intensity in U2OS cells after sgRNA transfection and 24 h of PDS (10 μM) treatment. The number of large RPA foci per cell is shown by a color scale. 1000 cells are shown for each condition. Results from one representative experiment are shown.
B. Mean percentage of cells with at least 6 large RPA foci, related to A. Data are represented as mean ± SEM (n = 4). One-way ANOVA followed by Dunnett's test was

performed comparing 4 mock treated samples, or 4 PDS treated samples. Test results comparing to the WT U2OS mock or PDS treated samples were shown.

C. Western blot in RPE1 cells 72 h after sgRNA transfection.
D. Scatter plots of total DNA content versus mean EdU intensity in RPE1 cells after sgRNA transfection and 24 h of PDS (5 μM) treatment. The number of large RPA foci per cell is shown by a color scale. 1000 cells are shown for each condition. Results from one representative experiment are shown.
E. Representative IF images of large RPA foci and telomere detection in WT and HLTF-KO U2OS cells. Arrowheads mark the telomere colocalizing large RPA foci.
F. Percentage of large RPA foci that colocalize with telomeres in WT and HLTF-KO U2OS cells. Data are represented as mean ± SEM (n=3).
G. Average number of large RPA foci colocalizing with telomeres per cell in WT and HLTF-KO U2OS cells. Data are represented as mean ± SEM (n=4).
H. Average percentage of telomere overlapping with γH2AX per cell in U2OS WT and HLTF-KO cells after 24 h of PDS (10 μM) treatment. Data are represented as mean ± SEM (n=3). All statistical tests in this figure are one-way ANOVA followed by Dunnett's test.

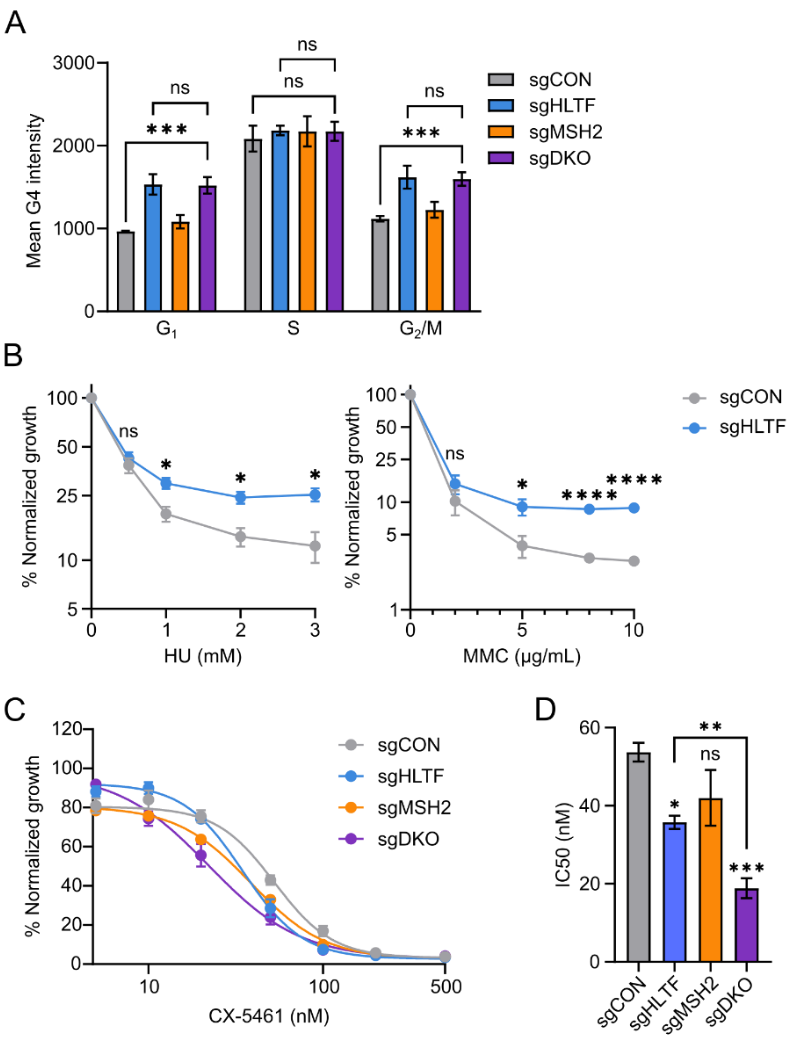

Figure S7. HLTF restrains replication fork progression and protects cells from DNA damage and growth defects in response to G4-stabilization. Related to Figure 7.

A. Mean G4 intensity in RPE1 cells, 72 h after sgRNA transfection. The median of the mean G4 intensity is used to calculate the mean ± SEM (n=5). Cells are transfected with sgRNAs against both HLTF and MSH2 in the sgDKO sample. T-test was performed.
B. Drug-response curve of HU and MMC in RPE1 cells after sgRNA transfection. Data are represented as mean ± SEM (n=4). T-test was performed.
C. Drug-response curve of CX-5461 in RPE1 cells after sgRNA transfection. In B and C, confluence was used as a readout after six days of treatment. Cells are transfected with sgRNAs against both HLTF and MSH2 in the sgDKO sample. Data are represented as mean ± SEM (n=4).
D. IC50 value of RPE1 cells in response to CX-5461 treatment after sgRNA transfection, as shown in C. Data are represented as mean ± SEM (n=4). ∗ $p < 0.05$; ∗∗∗ $p < 0.001$; ns, not significant by one-way ANOVA followed by Dunnett's test compared to sgCON. ∗∗ $p < 0.01$, by t-test.
